# PRMT5-regulated splicing of DNA repair genes drives chemoresistance in breast cancer stem cells

**DOI:** 10.1038/s41388-024-03264-1

**Published:** 2024-12-18

**Authors:** Matthew S. Gillespie, Kelly Chiang, Gemma L. Regan-Mochrie, Soo-Youn Choi, Ciara M. Ward, Debashish Sahay, Paloma Garcia, Roland Arnold, Clare C. Davies

**Affiliations:** 1 Department of Cancer and Genomic Sciences, University of Birmingham, Birmingham, B15 2TT UK; 2 Present Address: School of Cancer Sciences, University of Southampton, Southampton, SO16 6YD UK; 3 Present Address: Johnson & Johnson, 1400 McKean Rd, Spring House, PA 19002 USA

**Keywords:** Breast cancer, Homologous recombination, Methylation, Transcriptomics, Cancer stem cells

## Abstract

Breast cancer stem cells (BCSCs) are a rare cell population that is responsible for tumour initiation, metastasis and chemoresistance. Despite this, the mechanism by which BCSCs withstand genotoxic stress is largely unknown. Here, we uncover a pivotal role for the arginine methyltransferase PRMT5 in mediating BCSC chemoresistance by modulating DNA repair efficiency. Mechanistically, we identify PRMT5 as a major regulator of DNA damage response (DDR) gene splicing in BCSCs, particularly those integral to the Fanconi Anaemia and homologous recombination pathways, with PRMT5 inhibition synergising with chemotherapy to promote BCSC apoptosis. A comparison of BCSCs and their bulk cell progeny identified some shared (*ATM, DDX11, EXO1, FAN1, SLX4*) but many unique (*ATR, RAD17, RAD51D, RUVBL1*) PRMT5-dependent alternative DDR splicing events. Surprisingly, these skipped exons and retained intron events rarely lead to substantial gene expression repression, suggesting that PRMT5 inhibition predominantly results in nuclear detention of intron-containing transcripts and the production of non-canonical isoforms with compromised protein function. Since many genes within the same DDR pathway undergo deregulated splicing, this study thus reveals additional points of vulnerability and alternative combination drug strategies that could improve the therapeutic efficacy of PRMT5 inhibitors to promote BCSC eradication.

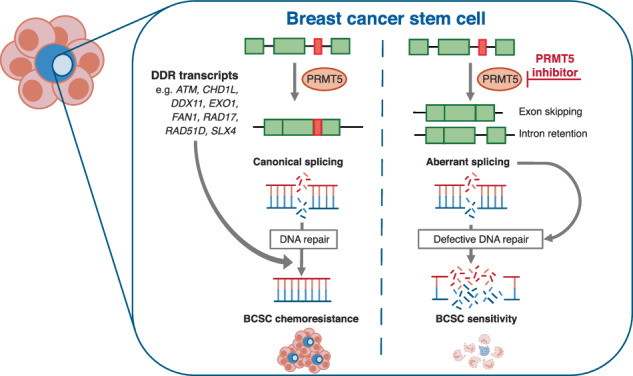

## Introduction

Breast cancer stem cells (BCSCs), or “tumour-initiating cells”, comprise a subpopulation of cancer cells within the tumour that have extended longevity, are able to self-renew and produce progeny that differentiate to form the heterogenous tumour mass. In contrast to the tumour bulk, BCSCs exhibit substantial resistance to conventional anticancer therapies [1], and are thus thought to be responsible for local regrowth and metastatic dissemination. Paradoxically, conventional chemotherapy increases the subcellular proportion of BCSCs [2]. Drug targeting this population is thus essential for achieving long-term clinical remission and improving patient survival.

Cancer stem cell (CSC) drug resistance can be attributed to a reduced cell cycle rate, upregulation of drug transporters, detoxifying enzymes and EMT-inducing genes, metabolic reprogramming, epigenetic re-wiring leading to activation of stemness signalling pathways and enhanced DNA repair capabilities [3]. The latter mechanism is particularly significant because most chemotherapy-induced toxicity is dependent on the induction of catastrophic DNA damage, particularly double strand breaks (DSBs). Indeed, the longevity of CSCs and their rapid proliferation compared to normal stem cells necessitate the maintenance of genome stability that is heavily reliant on homologous recombination (HR)-mediated DSB repair [4, 5]. For example, BCSCs isolated from triple negative breast cancer cell lines exhibit elevated levels of RAD50, a component of the MRN complex [6] whilst BCSCs isolated from patient-derived xenografts (PDXs) are enriched in a replication stress (RS) gene signature compared to bulk cells [7]. Despite this, the mechanisms underlying enhanced DNA repair in BCSCs following chemotherapy-induced DNA damage remain largely unexplored.

We have previously identified the arginine methyltransferase PRMT5, a protein associated with breast cancer progression, metastatic disease, and poor prognosis [8–14], as an important regulator of BCSC function and survival [15]. PRMT5 catalyses the majority of symmetric dimethylation (SDMA) in mammalian cells, and its association with cancer progression in both solid and haematological disease has prompted the rapid development of small molecule inhibitors that are now in clinical trials [16–20]. Interestingly, PRMT5 is also an important regulator of genome stability and DNA repair through multiple mechanisms. These include direct dimethylation (e.g. RUVBL1, FEN1, RAD9, TDP1, 53BP1) and epigenetic activation of DNA repair proteins involved in HR, the RS response and Fanconi Anaemia (FA) pathways [21–27]. Critically, whilst PRMT5 expression is generally elevated in cancer cells compared to non-transformed cells, its expression is further heightened within the BCSC population [15], suggesting that PRMT5 confers a survival advantage in this cell type.

Here, we demonstrate that BCSCs are more resistant to cisplatin and radiotherapy-induced cell death compared to the bulk tumour population, repair damaged DNA more efficiently than bulk cells, and that this is dependent on PRMT5 activity. Mechanistically, PRMT5 is required to maintain splicing fidelity of DNA repair transcripts, particularly those associated with the HR and FA pathways. Changes in differential splicing mediated by PRMT5 inhibition rarely correlate with substantial gene expression changes, implying that the splicing products are relatively stable, potentially yielding non-canonical isoforms or transcripts with detained introns. Combining PRMT5 inhibitors with chemotherapeutic agents that induce interstrand crosslinks (ICLs) and/or DSBs, or inhibitors targeting DNA repair proteins could therefore be an effective therapeutic strategy to eradicate chemoresistant BCSCs.

## Results

### BCSCs display resistance to DNA-damaging agents through enhanced DNA repair

**Fig. 1:**
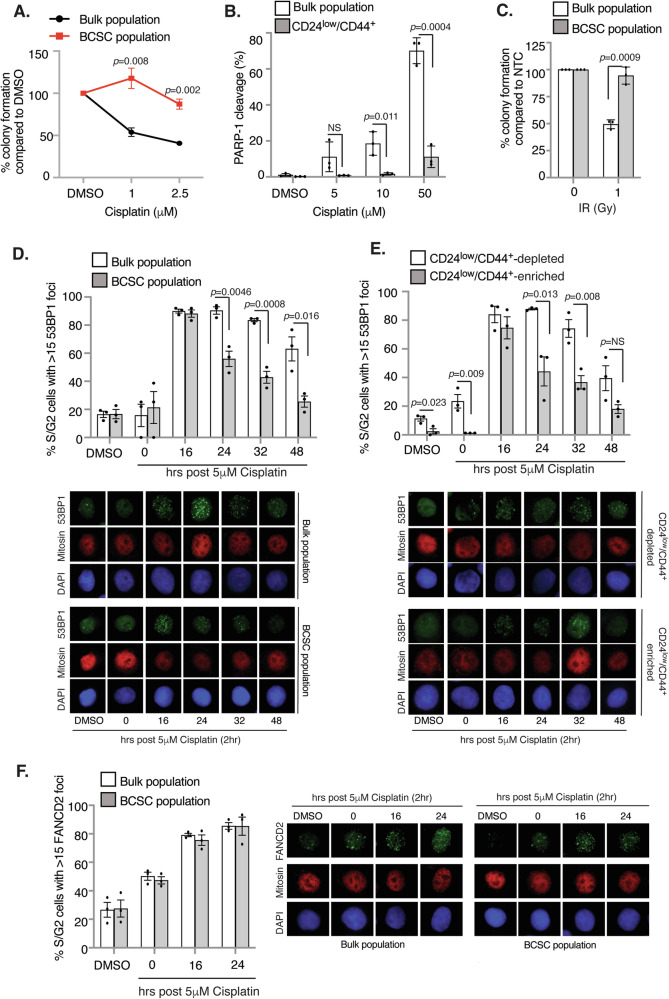
Breast cancer stem cells (BCSCs) are more chemoresistant than the bulk tumour population. **A** Colony survival analysis of cisplatin-treated bulk or AR BCSCs. **B** PARP1 cleavage assay of cisplatin-treated bulk or CD24^low^/CD44^+^ MCF7 BCSCs (mean ± SD). **C** Colony survival analysis of IR-treated MCF7 bulk or AR BCSCs (mean ± SD). 53BP1 foci analysis of cisplatin-treated (**D**) MCF7 AR or (**E**) CD24^low^/CD44^+^ BCSCs. Foci scored in mitosin-positive cells. **F** FANCD2 foci analysis of cisplatin-treated MCF7 bulk or AR BCSCs. Foci scored in mitosin-positive cells. Unless otherwise stated *n* = 3; mean ± SEM; Student’s *t* test (two-sided, equal variance).

Chemotherapy and radiotherapy promote cytotoxicity through the generation of DSBs, and increased DNA repair capacities and maintenance of genome stability has been linked to CSC chemo/radioresistance [1]. Although BCSCs isolated from mouse mammary tumours exhibit reduced sensitivity to ionising radiation (IR) and enhanced resolution of IR-induced DNA damage [28], few studies have comprehensively examined cisplatin chemoresistance in human BCSCs. To address this, we isolated BCSCs from the ER^+^ breast cancer cell line MCF7 using two methods: flow cytometry gating on ESA^+^CD24^low^/CD44^+^ cells [29, 30] and functional isolation by anoikis-resistance (AR) which is dependent upon survival in anchorage-independent conditions [31]. AR BCSCs cycle slower than bulk tumour cells, are more tumour-initiating in vivo (Supplementary Fig. S1A, B) and more resistant to cisplatin (Fig. 1A). Likewise, CD24^low^/CD44^+^ BCSCs are highly resistant to cisplatin, even at non-clinical doses (50 μM) (Fig. 1B). MCF7 BCSCs also displayed increased resistance to IR exposure (Fig. 1C).

Cisplatin forms covalent bonds with two adjacent purine bases, either on the same (intrastrand crosslinks) or opposite strands (interstrand crosslinks; ICLs). ICLs are cytotoxic, preventing DNA strand separation during DNA replication leading to the formation of one-ended DSBs. ICL repair thus involves the complex interplay between the Fanconi Anaemia (FA) and translesion synthesis (TLS) pathways, ultimately requiring homologous recombination (HR) for DSB resolution. To determine whether the increased cisplatin resistance in BCSCs was associated with enhanced DNA repair, we analysed DSB repair kinetics through the clearance of 53BP1 foci in late S/G2 cells, indicative of HR-mediated DSB resolution. Bulk tumour cells and both AR and CD24^low^/CD44^+^ BCSCs effectively sensed cisplatin-ICLs, mounting a comparably robust FA/DSB response following pulse cisplatin treatment (2 h), as evidenced by FANCD2 and initial 53BP1 recruitment respectively (Fig. 1D–F). Interestingly, BCSCs exhibited accelerated clearance of 53BP1 foci compared to bulk cells, with most cisplatin-induced DSBs being efficiently repaired within 48 h (Fig. 1D, E). Importantly, the dose and duration of cisplatin used for DNA repair kinetic analysis did not affect cell viability, implying that the reduction in 53BP1 foci was not due to enhanced apoptosis (Supplementary Fig. S2A). Similarly, IR-induced DNA damage and DSB formation, as determined by γH2AX and 53BP1 clearance respectively, was repaired more rapidly in AR BCSCs compared to the bulk population (Supplementary Fig. S2B, C). Furthermore, BCSCs display increased expression of key DSB repair (*ATM*, *EXO1*, *PNPK*) and FA genes (*FANCA*, *FANCI*, *RFWD3*) relative to bulk cells (Supplementary Fig. S2D). These findings support the hypothesis that a primary mechanism underlying chemoresistance in BCSCs is an enhanced DDR enabling efficient repair of chemo/radiotherapy-induced DNA lesions.

### PRMT5 promotes efficient DNA repair and mediates chemoresistance in BCSCs

PRMT5 promotes the proliferation, survival, and self-renewal of BCSCs [15], rendering it a tractable target for BCSC eradication. Indeed, in a breast cancer xenograft model, PRMT5 depletion led to a 12-fold reduction in BCSCs [15]. PRMT5 is also a major regulator of genome stability and HR-mediated DSB repair, with depletion or inhibition sensitising cancer cells to chemotherapeutic DNA-damaging agents including cisplatin and radiotherapy [20, 24, 32]. Given this, we hypothesised that PRMT5 activity contributes to cisplatin chemoresistance in BCSCs by promoting enhanced DNA repair capabilities.

**Fig. 2:**
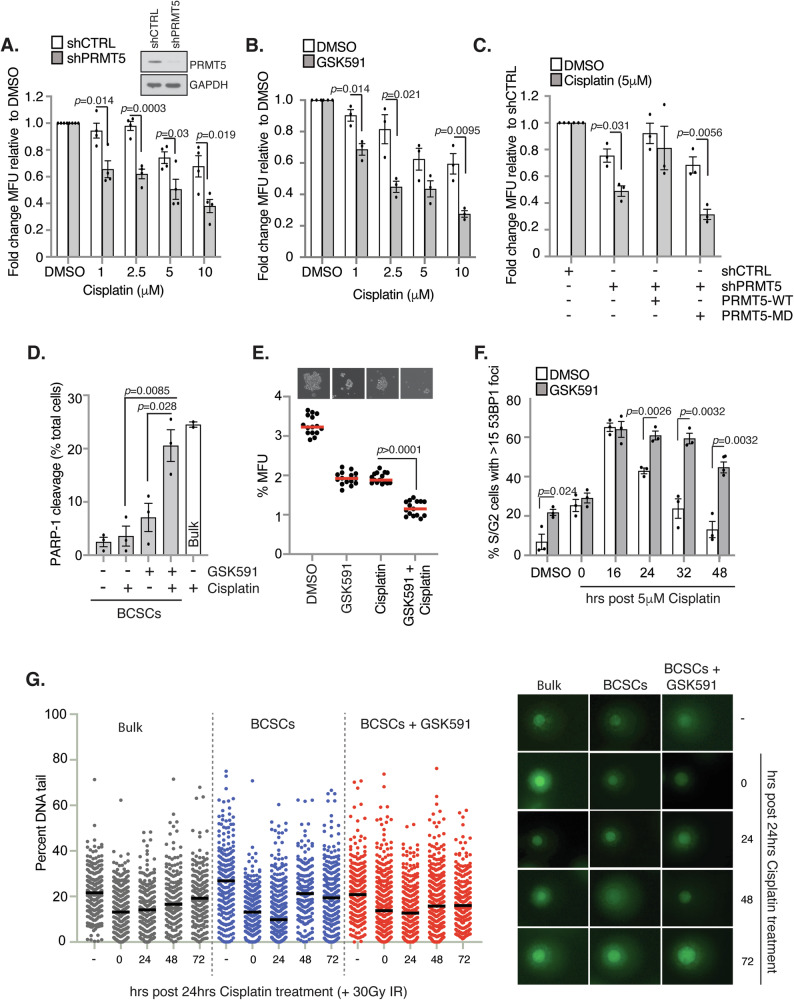
PRMT5 promotes cisplatin chemoresistance by regulating efficient DNA repair in BCSCs. **A** Mammosphere assay of cisplatin-treated MCF7-shCTRL or -shPRMT5 cells. **B** Mammosphere assay of cisplatin and GSK591-treated MCF7 cells. **C** Mammosphere assay of cisplatin-treated MCF7-shPRMT5 cells reconstituted with wildtype (WT) or methyltransferase dead (MD) PRMT5. **D** PARP1-cleavage assay of cisplatin and GSK591-treated AR BCSCs. White bar indicates bulk cells treated with 50 μM cisplatin. **E** Mammosphere assay of tumour-derived MCF7 cells treated with cisplatin and GSK591 ex vivo (*n* = 15). **F** 53BP1 foci analysis of cisplatin-treated AR BCSCs isolated from GSK591-treated MCF7 cells. Foci were scored in mitosin-positive cells. **G** Alkaline comet assay of MCF7 cells treated with GSK591 and cisplatin. AR BCSCs were isolated and treated with 30 Gy IR (bar = median). Unless otherwise stated *n* = 3; mean ± SEM; Student’s *t* test (two-sided, equal variance).

We found that shRNA-mediated depletion of PRMT5 increased sensitivity of BCSCs to cisplatin and IR by mammosphere assay, a measure of the survival and proliferation of BCSCs (Fig. 2A and Supplementary Fig. S3B). Crucially, PRMT5 methyltransferase activity was essential for chemoresistance because the selective PRMT5 inhibitor GSK3203591 [33, 34] (herein referred to as GSK591; Supplementary Fig. S3A) increased cisplatin and IR sensitivity in MCF7 and AU565 BCSCs (Fig. 2B, Supplementary Fig. S3C, D). To confirm the importance of the enzymatic activity, we established cell lines that enabled ectopic re-expression of wildtype (WT) or catalytically inactive PRMT5 (PRMT5-MD) after shRNA-mediated PRMT5 depletion (Supplementary Fig. S3E). As anticipated, PRMT5-MD, but not PRMT5-WT, sensitised MCF7 BCSCs to cisplatin (Fig. 2C). Critically, whilst GSK591 or cisplatin monotherapy induced low levels of apoptosis in BCSCs, combination treatment was highly effective in killing BCSCs akin to high-dose cisplatin monotherapy (Fig. 2D). Finally, to corroborate our findings from cell line-based experiments, MCF7 cells were subcutaneously implanted into NSG mice and ex vivo mammosphere assays performed. Whilst GSK591 and cisplatin monotherapy reduced mammospheres by approximately 40%, combination treatment was synergistic, further reducing BCSC survival (Fig. 2E). Taken together, these findings suggest that PRMT5 inhibition sensitises BCSCs to chemotherapy-induced cell death.

Given that BCSCs display an enhanced DDR and increased sensitivity to cisplatin or IR upon PRMT5 depletion or inhibition, we hypothesised that PRMT5 contributes to BCSC chemoresistance by promoting efficient DNA repair. We found that PRMT5 inhibition resulted in the persistence of 53BP1 foci in both AR and CD24^low^/CD44^+^ BCSCs following cisplatin-induced DSBs (Fig. 2F and Supplementary Fig. S3F). Similarly, PRMT5 activity was required for efficient repair of IR-induced DNA breaks in BCSCs (Supplementary Fig. S3G, H).

To directly examine the repair of cisplatin-ICLs, we conducted alkaline comet assays. ICLs crosslink the DNA duplex, preventing fragmentation of DNA at high dose irradiation. A low percentage tail moment indicates more ICLs (Supplementary Fig. S3I). BCSCs were able to resolve cisplatin-induced ICLs more rapidly than bulk cells (48 h), which was largely suppressed by GSK591 treatment (Fig. 2G). In summary, our data suggests that PRMT5 activity is required for repair of cisplatin and IR-induced DNA damage in BCSCs, and that PRMT5 inhibition leads to unresolved cytotoxic, chemotherapy-induced DNA damage.

### Overexpression of PRMT5 promotes enhanced DNA repair and chemoresistance in BCSCs

**Fig. 3:**
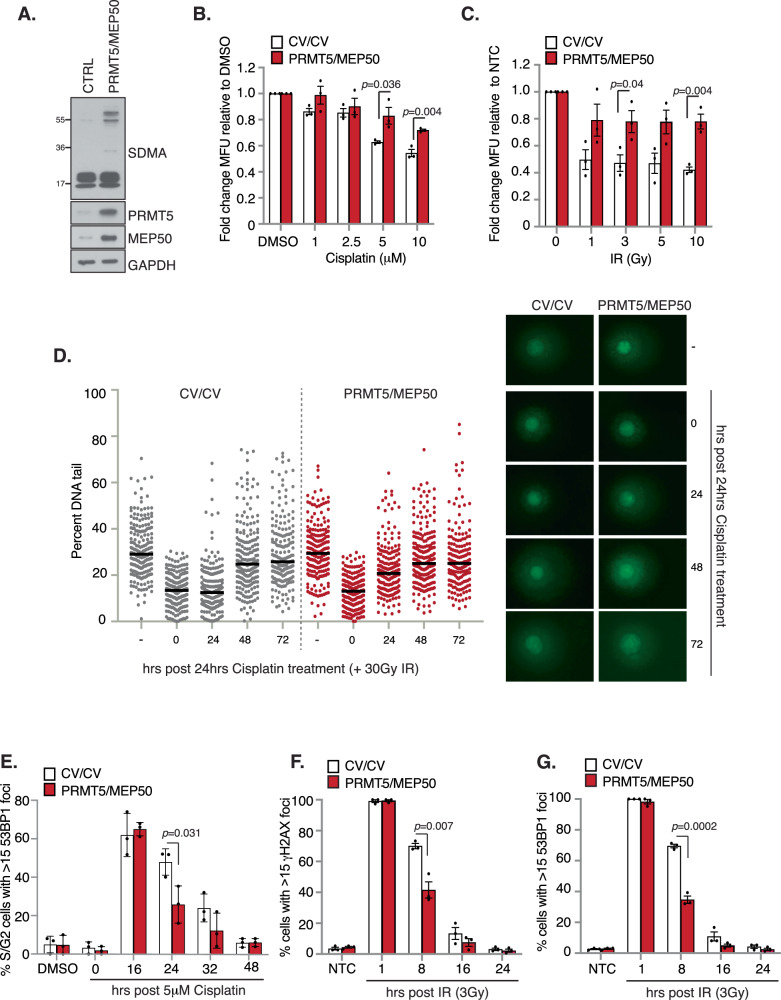
Overexpression of PRMT5 enhances DNA repair efficiency in BCSCs and confers increased chemoresistance. **A** Immunoblot of SDMA in MCF7 PRMT5/MEP50-overexpressing cells. Mammosphere assay of MCF7-CV (control vector) or PRMT5/MEP50 cells after (**B**) cisplatin or (**C**) IR. **D** Alkaline comet assay of AR BCSCs isolated from cisplatin-treated MCF7-CV or PRMT5/MEP50 cells (bar = median). **E** 53BP1 foci analysis of cisplatin-treated MCF7-CV or PRMT5/MEP50 cells (mean ± SD). **F** γH2AX foci and **G** 53BP1 foci analysis of IR-treated MCF7-CV or PRMT5/MEP50 cells. Unless otherwise stated *n* = 3; mean ± SEM; Student’s *t* test (two-sided, equal variance).

We have previously shown that BCSCs express higher levels of PRMT5 compared to bulk MCF7 cells and ectopic overexpression of PRMT5 and its essential cofactor MEP50 significantly increases BCSC frequency, self-renewal, and tumour initiation in vivo [15]. Intriguingly, mammosphere assays of BCSCs isolated from MCF7 cells co-overexpressing PRMT5/MEP50 exhibit heightened resistance to cisplatin and IR compared to control BCSCs (Fig. 3A–C), correlating with accelerated clearance of cisplatin-induced ICLs and 53BP1 foci (Fig. 3D, E), and increased efficiency in IR-induced DSB repair (Fig. 3F, G). These findings strongly suggest that elevated PRMT5 expression augments DNA repair efficiency in BCSCs contributing to chemoresistance.

### PRMT5 regulates splicing fidelity of DNA repair genes in BCSCs

PRMT5 is an important regulator of RNA splicing, methylating the Sm proteins SmB/B’, SmD1 and SmD3 which recruits SMN via its Tudor domain, enhancing constitutive spliceosome activity [35]. Given its involvements in splicing regulation in normal neuronal and haematopoietic stem cells [36, 37], and its recently reported contribution to splicing in glioblastoma stem cells (GSCs) [38], we hypothesised that PRMT5 mediates chemoresistance in BCSCs through RNA splicing mechanisms.

**Fig. 4:**
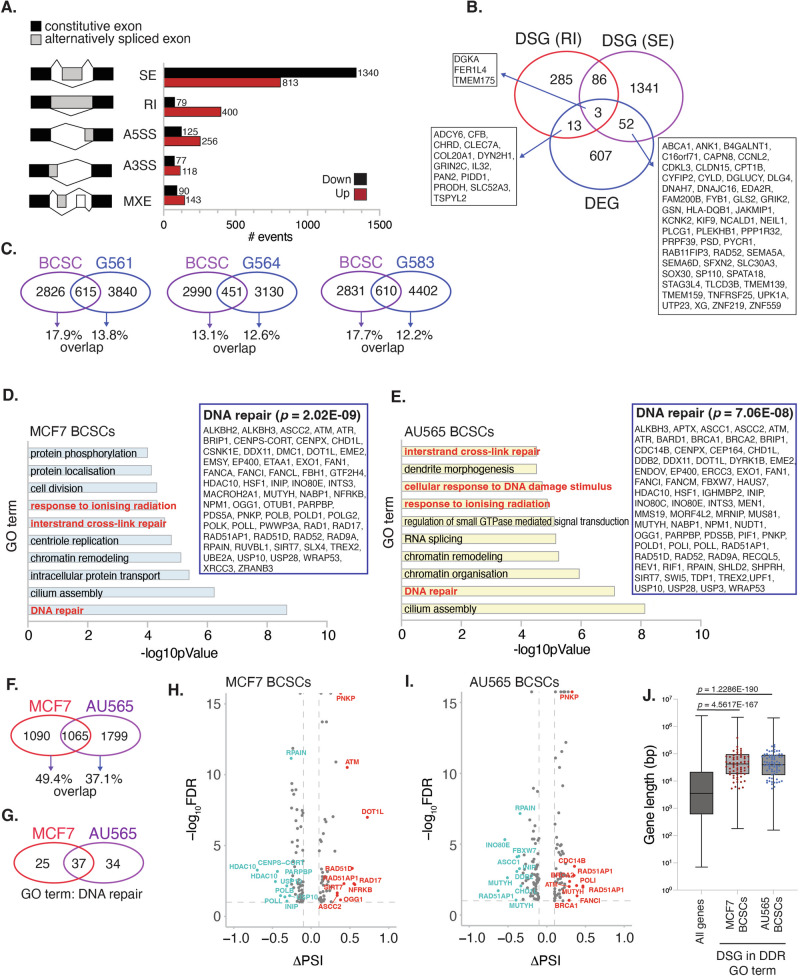
Inhibition of PRMT5 in BCSCs leads to defective splicing of DNA repair transcripts. **A** Proportion of significant differential splicing events (ΔPSI > 0.1; FDR < 0.1) in GSK591-treated MCF7 AR BCSCs. SE Skipped Exon, RI Retained Intron, A5SS Alternative 5’ Splice Site, A3SS Alternative 3’ Splice Site, MXE Mutually Exclusive Exon, *n* = 3. **B** Overlap of differentially spliced genes (DSGs) with SE/RI events and differentially expressed genes (DEGs) in GSK591-treated MCF7 AR BCSCs. **C** Comparison of GSK591-induced differential splicing events in MCF7 AR BCSCs and three glioblastoma (GBM) cancer stem cell (CSC) lines (G561, G564, G583). GBM CSC data from ref. [38]). GO of significant DSGs in GSK591-treated (**D**) MCF7 AR BCSCs and (**E**) AU565 AR BCSCs. Inset box displays genes identified in the GO:DNA repair. Top 10 enriched terms are shown. Comparison of GSK591-induced (**F**) DSGs and (**G**) GO:DNA repair DSGs in MCF7 and AU565 AR BCSCs. **H** Volcano plot of DSGs identified within the GO:DNA repair (ΔPSI > 0.1; FDR < 0.1) from **H** MCF7 BCSCs and **I** AU565 BCSCs. **J** Box plot of gene length of DSGs in MCF7 and AU565 AR BCSCs compared to all genes. Red and blue dots represent genes belonging to the GO:DNA repair for MCF7 and AU565, respectively.

To investigate this, RNA-seq analysis of GSK591-treated MCF7 AR BCSCs was performed. Significant differential splicing events (DSEs) (FDR < 0.1 and ΔPSI > 0.1 (>10% of total transcripts)) were determined by rMATS [39]. Our analysis revealed 3441 DSEs across 2155 genes following PRMT5 inhibition in AR BCSCs (Fig. 4A). Closer examination shows that GSK591 promotes enrichment of skipped exons (SE) and retained introns (RI), as observed in other cell types [37, 38, 40, 41]. Specifically, we observed that 62.6% of DSEs were SEs (an event that includes either aberrant retention or exclusion of the specific exon after GSK591 treatment) and 13.9% were RI events. Whilst many genes experienced only one type of splicing event, others exhibited both RI and SE events, suggesting that PRMT5 can influence multiple splicing events within a single transcript (Fig. 4B).

To determine whether aberrant splicing events detected in BCSCs were common to other CSCs, we compared our data with published RNA-seq datasets from three GSK591-treated (1 μM, 3 days) glioblastoma stem cell lines (GSCs) [38]. Whilst the total number of aberrant splicing events were similar across the four cell lines, the degree of overlap in specific events was low (Fig. 4C), suggesting that PRMT5 splicing occurs across different genes in a CSC type-specific manner. Thus, whilst the type of splicing event regulated by PRMT5 is common across cell types, the genes which undergo differential splicing are diverse. Supporting this, Gene Ontology (GO) analysis of differentially spliced genes (DSGs) showed that “cell cycle”-related GO terms were most significantly enriched in GSCs [38], whereas MCF7 BCSCs were enriched for “DNA repair”, “interstrand crosslink repair” and “responses to ionising radiation” (Fig. 4D). Analysis of GSK591-induced splicing events in BCSCs derived from Her2^+^ AU565 cells also identified “DNA repair” as one of the most enriched GO terms (Fig. 4E). Interestingly, the two breast cancer subtypes displayed greater overlap in DSGs (49.4% and 37.1%) than MCF7 BCSCs with GSCs (12.2% to 17.9%) (Fig. 4F) with further examination of the “DNA Repair” grouping identifying 37 commonly spliced DNA repair genes (Fig. 4G and Supplementary Fig. S4A). Indeed, numerous GSK591-induced splicing events were consistent between the two subtypes of BCSCs occurring at the same genomic location (Supplementary Table 1). These findings suggest that PRMT5 significantly impacts on the splicing of DNA repair genes in BCSCs, even targeting the same splicing event across different breast cancer subtypes. Further analysis also revealed that DSEs occurred in several genes within the ICL and HR DNA repair pathways (Fig. 4H, I), suggesting that aberrant splicing of multiple genes within a single DNA repair pathway could profoundly impact the ability of a cell to orchestrate effective repair. Interestingly, we found that GSK591-mediated deregulated splicing events in both MCF7 and AU565 AR BCSCs were enriched in long transcripts, with our differentially spliced DNA repair genes positioned towards the top end of this group (Fig. 4J), implying that PRMT5 activity primarily targets the processing of long transcripts.

**Fig. 5:**
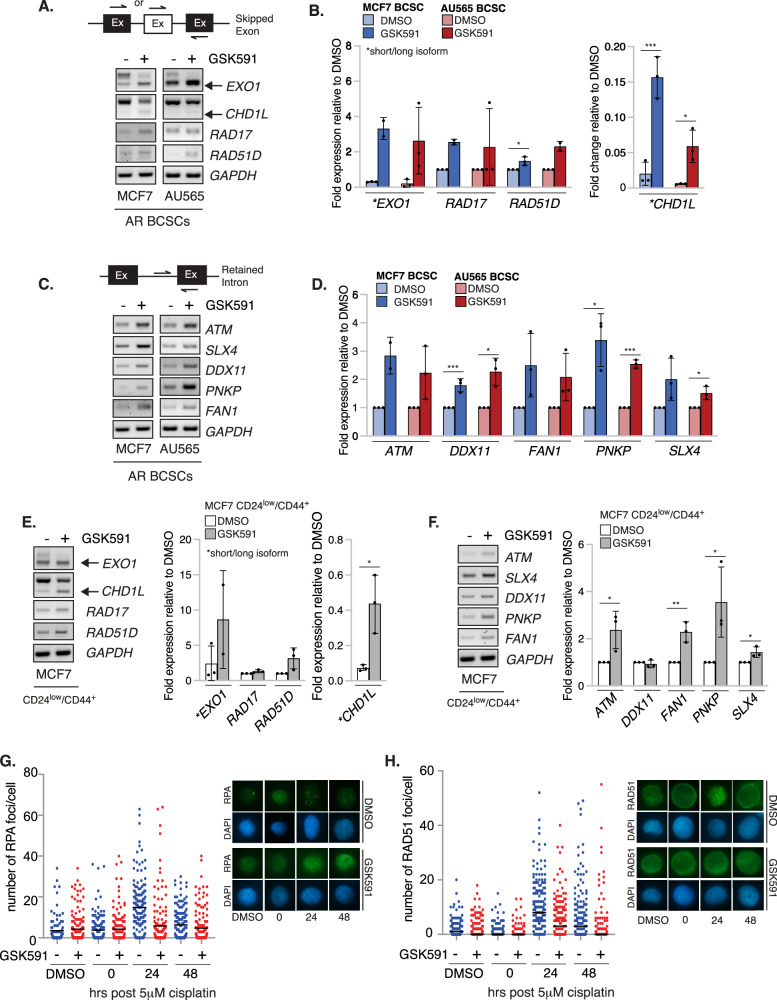
PRMT5 regulates the canonical splicing of *EXO1, RAD17, RAD51D, CHD1L, ATM, DDX1, FAN1, PNKP*, and *SLX4* in MCF7 and AU565 BCSCs. **A** RT-PCR validation of SE splicing events in GSK591-treated MCF7 and AU565 AR BCSCs. Representative image of *n* = 3. **B** Densitometry quantification of data in **A** and Supplementary Fig. S4B (*n* > 2). **C** RT-PCR validation of RI splicing events in GSK591-treated MCF7 and AU565 AR BCSCs. Representative image of *n* = 3. **D** Densitometry quantification of data in **C** and Supplementary Fig. S4B (*n* > 2). **E** RT-PCR validation and quantification of SE splicing events in GSK591-treated MCF7 CD24^low^/CD44^+^ BCSCs (*n* = 3; mean ± SD). **F** RT-PCR validation and quantification of RI splicing events in GSK591-treated MCF7 CD24^low^/CD44^+^ BCSCs (*n* = 3; mean ± SD). **G** RPA and **H** RAD51 foci analysis of cisplatin and GSK591-treated MCF7 AR BCSCs (*n* = 3; >50 cells counted per replicate, bar=median).

Using samples derived independently from those analysed by RNA-sequencing, we validated a subset of GSK591-induced DSEs in genes involved in ICL/HR repair in both MCF7 and AU565 AR BCSCs. We also included *ATM* and the NHEJ/BER factor *PNKP* whose mRNA transcripts are known to undergo splicing in a PRMT5-dependent manner [26, 41]. Using primers that capture either the retained intron or spliced-out exon, we verified that GSK591 induced the formation of shorter isoforms of *EXO1* and *CHD1L*, along with the inclusion of a non-canonical exon in the *RAD17* and *RAD51D* transcripts (Fig. 5A, B, Supplementary Table 2, Supplementary Fig. S4B, S4D). Similarly, RI events observed in *ATM, SLX4, DDX11, PNKP* and *FAN1* were independently confirmed (Fig. 5C, D, Supplementary Fig. S4B, S4E). Many of these differential splicing events induced by PRMT5 inhibition were also observed in MCF7 CD24^low^/CD44^+^ BCSCs indicating that these events are present in BCSCs irrespective of how they are isolated (Fig. 5E, F, Supplementary Fig. S4C). Importantly, these splicing changes in ICL repair genes correlated with defective HR as treatment of BCSCs with GSK591 significantly reduced RPA and RAD51 foci formation, indicating reduced end-resection and RAD51 nucleofilament formation respectively (Fig. 5G, H). Our data thus suggest that the disruption of DDR transcript splicing results in inefficient ICL/HR repair that could be exploited to sensitise BCSCs to DNA damaging agents.

### Alternative splicing events induced by PRMT5 inhibition in BCSCs rarely correlate with substantial alterations in gene expression

**Fig. 6:**
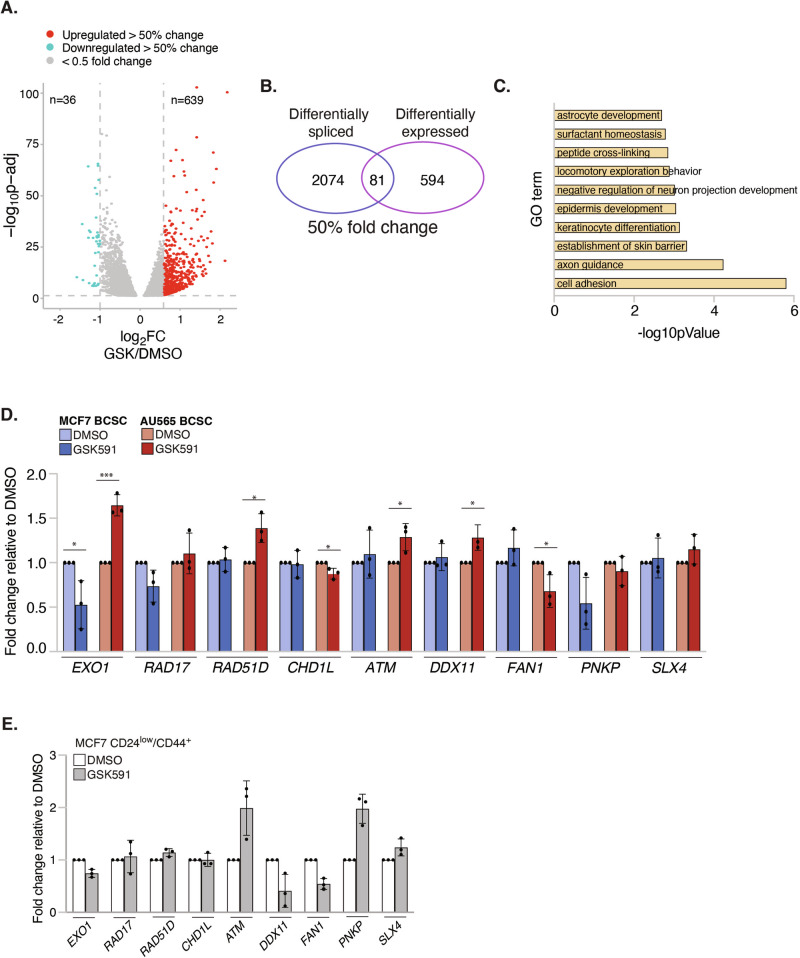
GSK591-induced splicing alterations results in minor gene expression changes. **A** Volcano plot of differentially expressed genes (DEGs) (*p*-adj <0.05) in GSK591-treated MCF7 AR BCSCs. Red and blue dots signify genes whose expression increased or decreased by 50% respectively (*n* = 3). **B** Venn diagram of DEGs (*p-*adj <0.05; >50% fold change) and DSGs (ΔPSI > 0.1; FDR < 0.1) in GSK591-treated MCF7 AR BCSCs. **C** GO of GSK591-induced DEGs (*p-*adj <0.05; >50% fold change). **D** qPCR validation of gene expression changes in GSK591-treated MCF7 and AU565 AR BCSCs. **E** qPCR validation of gene expression changes in GSK591-treated MCF7 CD24^low^/CD44^+^ BCSCs. Unless otherwise stated *n* = 3; mean ± SD; Student’s *t* test (two-sided, equal variance).

Changes in splicing often impact transcript levels and overall gene expression. For example, intron retention is predicted to lead to premature termination codon (PTC) recognition and nonsense-mediated decay (NMD). We found that GSK591 treatment led to 5995 significant changes in gene expression (*p*-adj <0.05; Fig. 6A). To triage our list to those that are more likely to have biological impact, we focused on genes with >50% fold change following GSK591 treatment. Using this criterion, we identified a modest 675 genes with differential gene expression (DEGs), of which only 36 genes were downregulated (Fig. 6A, B, Supplementary Table 3). Our data therefore suggests that PRMT5 predominantly represses gene expression, presumable via H4R3 methylation [42–44]. GO analysis highlighted “cell adhesion” as the most significantly enriched GO term for GSK591-dependent DEGs (Fig. 6C). Interestingly, overlap of DEGs with >50% change in expression and DSGs revealed that only 3.76% of transcripts displayed both significant changes in splicing and gene expression (Fig. 6B). Further examination of RI and SE events in correlation with gene expression changes revealed that only 16/387 genes with retained introns and 55/1482 genes with skipped exons underwent >50% change in expression (Fig. 4B). This was corroborated by independent qPCR analysis of PRMT5-spliced DDR genes which showed that GSK591 had little impact on total mRNA levels (Fig. 6D, Supplementary Table 4), except for *RAD51D, ATM* and *DDX11* which was elevated in AU565 AR BCSCs, but not MCF7 AR BCSCs. Interestingly, GSK591 induced skipping of *EXO1*-Exon 3 in both AU565 and MCF7 AR BCSCs causing both elevated and reduced *EXO1* expression respectively (Fig. 6D). Furthermore, total mRNA of DDR genes with RIs was largely unchanged or in the case of *ATM* and *DDX11* in AU565 BCSCs, surprisingly elevated (Fig. 6D). Similar analysis in MCF7 CD24^low^/CD44^+^ BCSCs also showed that differential exon usage in *EXO1* and *CHD1L* after GSK591 did not significantly change total mRNA levels. In fact, *ATM* and *PNKP* transcript levels were increased despite GSK591 inducing a RI event (Fig. 6E). Collectively, our analysis of transcript levels of GSK591-treated BCSCs originating from two distinct breast cancer molecular subtypes suggests that non-canonical splicing events do not substantially impact the quantity of a targeted transcript but instead alter the balance between canonical and alternatively spliced products. Finally, the similarities observed between AR and CD24^low^/CD44^+^ MCF7 BCSCs imply the existence of shared PRMT5-dependent mechanisms across both BCSC populations.

### Inhibiting PRMT5 activity deregulates splicing in the bulk tumour population

**Fig. 7:**
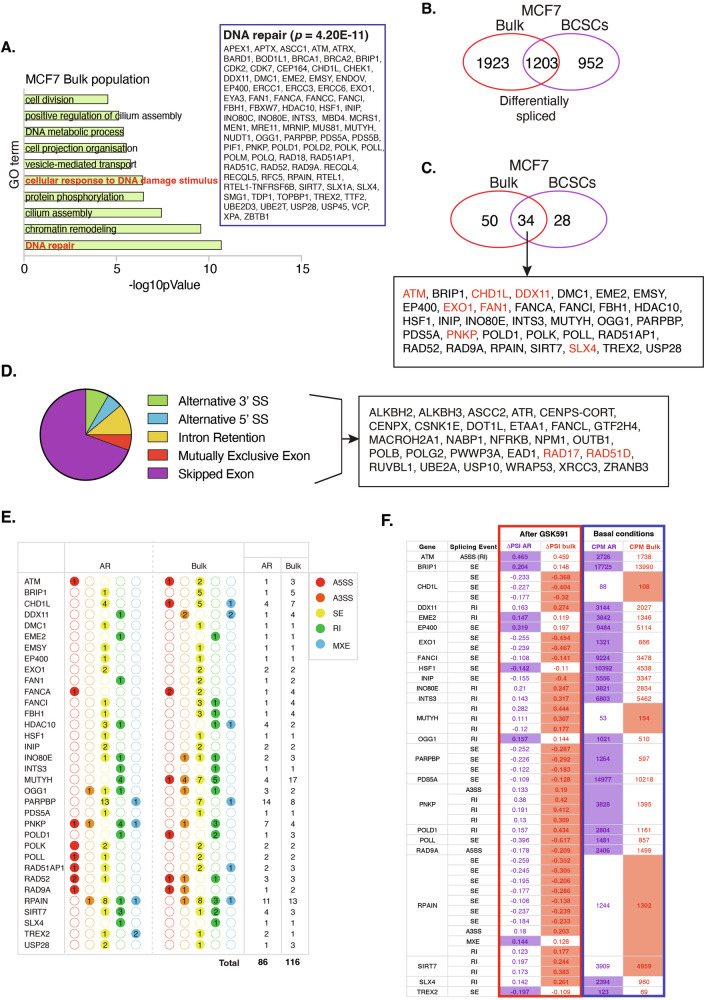
GSK591-mediated splicing changes in BCSCs partially overlap with those occurring in the bulk MCF7 cell population. **A** GO of GSK591-induced DSGs (ΔPSI > 0.1; FDR < 0.1). Inset box displays genes identified in the GO:DNA repair. Data from *n* = 2. Venn diagram displaying overlap between (**B**) DSGs and (**C**) GO:DNA repair DSGs in GSK591-treated MCF7 bulk and AR BCSCs (ΔPSI > 0.1; FDR < 0.1). Inset box displays DSGs common to both cell populations; red = independently validated. **D** Distribution of BCSC-specific, GSK591-mediated differential splicing events (ΔPSI > 0.1; FDR < 0.1) (28 gene set). Red = independently validated. **E** Table depicting the type (coloured circles) and number (inside circle) of splicing events in DSGs following GSK591 treatment in both MCF7 bulk and AR BCSCs. A5SS alternative 5’ splice site, A3SS alternative 3’ splice site, SE skipped exon, RI retained intron, MXE mutually exclusive exon. **F** Table showing the identical splicing events in DSGs in MCF7 bulk and AR BCSCs. Column 3 and 4 (grey box) show ΔPSI of identical DSEs after GSK591 treatment (shaded boxes indicate the higher values between bulk and AR BCSCs). Column 5 and 6 (blue box) indicate transcript levels of each DDR gene (counts per million) from DMSO controls (shaded boxes indicate the higher values between bulk and AR BCSCs).

So far, our focus has been restricted to the effects of GSK591 on splicing events within BCSCs. To determine whether these are BCSC-specific, we conducted RNA-sequencing of GSK591-treated bulk MCF7 cells. Our analysis showed 3126 DSGs in MCF7 bulk cells (5450 events) (ΔPSI > 0.1) with “DNA repair” being the most enriched GO term (Fig. 7A). Despite this similarity, 44.2% of splicing events in BCSCs were distinct (Fig. 7B) implying that PRMT5 does not regulate the same genes in both populations. Regarding DDR genes, whilst some transcripts such as *ATM, CHD1L, EXO1, FAN1, PNPK* and *SLX4* were common between cell types (Fig. 7C), others such as *ATR, DOT1L, RUVBL1*, *USP10*, *RAD17* *and* *RAD51D* were specific to the BCSCs (Fig. 7D). A number of these were validated by RT-PCR (Supplementary Fig. S5A–D). These findings emphasise PRMT5 as a global regulator of DDR transcript splicing in MCF7 cells, but also indicate specificity in BCSCs that could confer additional chemoresistance properties.

Given that PRMT5 levels are higher in BCSCs compared to bulk cells, we considered whether this would enable BCSCs to withstand splicing alterations to a higher degree than bulk cells, thereby explaining why bulk cells are more responsive to chemotherapy. We observed that of the 34 differential spliced genes induced by PRMT5 inhibition in both bulk and BCSCs (intersect in Fig. 7C), 14 had a higher number of individual differential splicing events (DSEs) in the bulk cells compared to BCSCs, 13 had the same number, and only 7/34 had more in BCSCs compared to bulk, implying that generally, DNA repair gene splicing is more sensitive to PRMT5 inhibition in bulk cells compared to BCSCs (Fig. 7E). Next, we identified 44 annotated identical splicing events occurring in the 34 differentially spliced DNA repair genes that overlap between MCF7 bulk and BCSCs and examined the degree of splicing changes after GSK591, as determined by ΔPSI. 36/44 identical splicing events showed a greater ΔPSI in bulk versus BCSCs after GSK591 (Fig. 7F, red boxed columns), but overall transcript levels of the DDR gene itself were higher in BCSCs compared to bulk cells (Fig. 7F, blue boxed columns). Collectively, these observations suggest that: (1) splicing of DDR genes in bulk cells is more sensitive (relative to number of DSE and magnitude of change) to PRMT5 inhibition; and (2) bulk cells express lower levels of DNA repair transcripts and are thus more susceptible to the effects of PRMT5 inhibition on splicing fidelity. Consequently, our data support the notion that elevated expression of DDR transcripts coupled with high PRMT5-mediated splicing fidelity in BCSCs leads to enhanced chemoresistance in this cell type.

### SRSF1 methylation does not regulate the splicing of selected DDR transcripts

To explore the mechanism by which PRMT5 regulates splicing in BCSCs, we focused on the RNA-binding protein SRSF1 because of its association with breast cancer, its ability to regulate cisplatin sensitivity, and that PRMT5-dependent methylation regulates splicing in AML cells [41, 45–47]. Whilst we found that siRNA-mediated knockdown or overexpression of SRSF1 in MCF7 cells regulated BCSC survival and cisplatin sensitivity via mammosphere assays (Supplementary Fig. S6A–C), neither depletion of SRSF1, nor expression of a methyl-deficient SRSF1 protein where R93, R97 and R109 are mutated to lysine [41], could phenocopy the splicing changes observed by GSK591 treatment in *EXO1* and *CHD1L* transcripts (Supplementary Fig. S6D). Whilst depletion of SRSF1 did lead to an RI event in SLX4 similar to that induced by GSK591, this is largely SRSF1-methyl independent. Taken together, whilst the significance of methyl-SRSF1 for splicing in BCSCs on a global level has yet to be determined, our data suggest that SRSF1 may not be the principal RBP that is methylated by PRMT5 that regulates splicing of DDR transcripts in this cell type.

## Discussion

BCSCs are a rare population of breast tumour cells that are relatively refractory to conventional therapies and are thus a major clinical concern, with residual BCSC activity following first line therapy a known driver of tumour regrowth and metastasis [2]. We previously showed that PRMT5 regulates BCSC function and survival via the epigenetic regulation of gene expression [15]. In this study, we expand on the importance of PRMT5 within the BCSC compartment to include the control of another key CSC trait, chemoresistance. Mechanistically, we find that PRMT5 is required to maintain the splicing fidelity of key HR and FA pathway genes enabling BCSCs to mount an efficient DDR against chemotherapy. Supporting this, overexpression of PRMT5 is sufficient to drive effective DNA repair and cisplatin resistance in BCSCs, whilst PRMT5 inhibition synergises with DNA damaging chemotherapies to promote BCSC cell death. Collectively, our findings propose that PRMT5 facilitates the accurate and timely processing of chemotherapy-induced DNA damage and that combining PRMT5 inhibitors with DNA damaging agents, or inhibitors to DDR proteins whose splicing is deregulated, could be an effective strategy to promote BCSC eradication leading to long-term patient survival.

PRMT5 directly methylates DNA repair proteins such as RUVBL1, FEN1 and RAD9 [20, 22–24]. Whilst we cannot rule out that these events also occur in BCSCs, our finding that “DNA Repair” was the most significantly enriched GO term for DSGs clearly demonstrates that BCSCs are particularly dependent on PRMT5 activity for DDR gene splicing and that this is a major mechanism by which PRMT5 drives chemoresistance in BCSCs. Interestingly, this contrasts with findings by Sachamitr et al. [38] in GSCs treated with GSK591 where cell cycle/mitotic cell cycle were identified as their top-ranked GO. Indeed, only nine DDR transcripts (*RPAIN, CHD1L, SIRT7, NFKRB, PNKP, MUTYH, GTF2H4, EP400, DDB2*) are common to both our BCSC datasets and the GSC dataset [38], highlighting the specificity of PRMT5 in regulating different genes within distinct CSCs. PRMT5 thus appears to regulate splicing in cancer stem cells in a tissue-dependent manner, with BCSCs potentially reliant upon a higher fidelity of DNA repair transcript splicing to maintain genome stability, longevity, and chemoresistance compared to GSCs.

Deregulated splicing induced by PRMT5 inhibition/depletion has been correlated with extensive changes in gene expression [26, 36–38, 48], however most of these studies considered statistically-defined significant changes rather than those beyond a predefined threshold. Although we identified thousands of significant gene expression changes after GSK591 treatment in BCSCs, applying a 50% fold change threshold to identify those that are more likely to have biological impact reduced this to just 675 transcripts. Notably, most of these transcripts (95%) exhibited increased expression after GSK591 treatment implying that PRMT5 predominantly acts to suppress gene expression which is consistent with its role in histone H4R3 symmetric dimethylation [42–44]. Consequently, our findings suggest that GSK591-induced splicing changes in BCSCs generally lead to an imbalance in productive transcripts rather than a depletion of total mRNA levels. In the case of SE and MXE events, this suggests that GSK591 promotes the generation of stable non-canonical isoforms, as supported by our validation of SE events without concurrent alterations in gene expression for *RAD17, RAD51D*, and *CHD1L* transcripts. Even more intriguingly, only 16/387 RI events displayed a 50% change in gene expression. This correlates well with results from THP-1 AML cells after *PRMT5* knockdown where only 10/321 RI events showed a corresponding change in gene expression [41], suggesting that impaired PRMT5 activity leading to non-spliced introns manifest as a detained intron (DI) event. DI are an evolutionary conserved set of mRNAs thought to occur due to reduced splicing kinetics at specific intron/exon junctions as most pre-mRNA processing proceeds normally. Significantly, despite 99.5% of introns containing an in-frame PTC, transcripts containing DI are not typically subjected to NMD because they are retained in the nucleus and not exported to the cytoplasm where NMD occurs [49, 50]. These DI events are not random and are often enriched in transcripts encoding for RNA metabolism and DNA repair proteins [50], acting as a reservoir that can rapidly undergo completion of splicing to rapidly increase protein levels. Consistent with this, DNA damage increases the rate of DI removal in the BRCA1-interacting protein *BCLAF1* [50].

More recently, PRMT5 inhibition has been linked to a defective replication stress response through the downregulated protein levels of ATR, which was attributed to reduced transcription rather than defective splicing [51]. In contrast, we see significant alteration in *ATR* splicing in both MCF7 and AU565 BCSCs but no alteration in mRNA levels. Likewise, although Li et al., demonstrated decreased protein levels of ATM and CHD1L after PRMT5 inhibition, our transcript analysis revealed differential splicing accompanied by either no change, or unexpectedly, an increase in mRNA levels. Although the proteomic data was collated from bulk cells from 12 different cancer cell lines originating from the breast, ovary, liver and pancreas, and we have not examined protein levels of DDR genes that undergo differential splicing after GSK591 in BCSCs, this does suggest that the mechanism by which PRMT5 regulates protein expression and function is divergent dependent on cancer origin and cell type. Together this highlights a multifaceted role for PRMT5 in protein expression that is highly context specific.

The splicing landscape is a highly understudied area of CSC biology even though alternative splicing signatures can distinguish between normal and malignant AML progenitor cells [52].The precise mechanism by which PRMT5 directs DDR gene splicing events in BCSCs remains to be fully elucidated. PRMT5 methylates snRNPs such as SNRPB (Sm-B/B’) and SNRPD3 (Sm-D3) and this is required for the post-transcriptional maturation of transcripts with retained introns. However, given that Sm-B/B’ and Sm-D3 regulate global RNA processing, additional levels of splicing control must occur in a cell type-dependent manner, for example methylation of RBPs that are upregulated in breast cancer [53, 54]. Given that SRSF1 has been linked to breast cancer and is methylated by PRMT5 [41, 54], we posited that SRSF1 may be one RBP that is methylated by PRMT5 contributing to splicing fidelity in BCSCs. Whilst we found that SRSF1 might be partially required for the proliferation of BCSCs, ectopic re-expression of methyl-deficient SRSF1 in SRSF1-depleted cells failed to phenocopy the effects of GSK591 on *EXO1, ATM, CHD1L* and *SLX4* splicing in BCSCs. Hence, the mechanisms by which PRMT5 contributes to cell-type and stimulus-specific splicing events is still largely unknown.

While certain splicing events, including those validated in our study (*ATM, CHD1L, DDX11, EXO1, FAN1, PNKP, SLX4*) are common to bulk tumour cells and BCSCs, numerous events are notably enriched in BCSCs, both providing new opportunities to identify combination PRMT5 inhibitor approaches that maximise the potential of PRMT5-directed therapy. This is especially significant for breast cancer patients as *MTAP* deletion, a trait conferring synthetic lethality with single agent second-generation PRMT5 inhibitors is rarely observed [55–59]. Furthermore, initial xenograft models suggest that first-generation PRMT5 inhibitors effective in *MTAP*-competent genetic backgrounds are cytostatic rather than cytotoxic because tumours regrew after treatment withdrawal [16]. Our observation that PRMT5 activity targets DDR gene splicing not only offers mechanistic insight into how PRMT5 inhibitors synergise with DNA-damaging chemotherapies to trigger BCSC apoptosis, but also identifies novel therapeutic strategies capitalising on the HR deficiency induced by PRMT5 inhibitors in HR proficient BCSCs [6, 60]. This strategy could prove particularly potent when aberrant splicing affects multiple genes within the same pathways, such as *RAD17, FANCA, FANCI, FANCL, FAN1, SLX4* and *ATR*. For instance, RAD17 plays a crucial role in activating the ATR signalling pathway following the detection of ICLs by the FA core complex, achieved through its interaction with the 9-1-1 clamp loading complex and TOPBP1 [61]. Consequently, a combination of PRMT5 and ATR inhibitors could prove particularly toxic to BCSCs. Similarly, the RAD51 paralogue RAD51D, acting in concert with BCDX2, contributes to RAD51 nucleofilament formation at damaged sites and regulates HR [62, 63]. Interestingly, GSK591 promoted the inclusion of Exon 3 that codes for *RAD51D* isoform 6 that incorporates an additional region adjacent to the Walker A motif. This is known to impair HR-mediated DSB repair, suggesting that PRMT5 inhibitors could synergise with RAD51 inhibitors [64, 65].

Our study has uncovered the critical role of PRMT5 activity in regulating the canonical splicing of DDR transcripts within the chemoresistant BCSC population. Combining PRMT5 inhibitors with DNA-damaging chemotherapies and/or innovative DDR inhibitors targeting could widen the therapeutic window that promotes effective BCSC and bulk tumour cell eradication whilst minimising unwanted toxicities. Future studies elucidating how PRMT5 directs specific splicing events in BCSCs has the potential to provide valuable biological insights into the survival mechanisms of this enduring tumour-initiating cell subpopulation.

## Methods

### Cell lines and cell culture

MCF7 and AU565 breast cancer cell lines were maintained in Dulbecco’s Modified Eagle’s Medium (DMEM; Merck Life Sciences) supplemented with 10% heat-inactivated foetal bovine serum (FBS; Gibco), 5U/ml penicillin and 1 mg/ml streptomycin (Merck Life Sciences). For suspension culture, cells were maintained in serum-free DMEM/F12 (without phenol red) (Gibco) supplemented with 1X B-27 Supplement minus vitamin A (Invitrogen) and 20 ng/ml human recombinant epidermal growth factor (Gibco). MCF7 stably expressing shPRMT5 in combination with ectopic wildtype and catalytic dead (G367A/R368A) PRMT5 have been described [15]. MCF7 cells co-overexpressing PRMT5 and MEP50 have been described [15]. The siLUC sequence is 5’-CGUACGCGGAAUACUUCGA-3. siSRSF1 sequence (5’-GCAACCACGAAACCUGUAAUA-3’), although annotated as targeting the 3’UTR, substantially base-pairs with the 3’ coding sequencing leading to depletion of ectopic SRSF1. MCF7-Flag-SRSF1 WT and 3R-K overexpressing cells were thus generated by cloning siSRSF1-resistant human SRSF1 WT or R93K, R97K, R109K [41] into pHIV-zsGreen lentiviral vector.

### Isolation of anoikis-resistant BCSCs and drug treatments

MCF7 cells were plated onto polyHEMA-coated 150 mm dishes at a density of 900 cells/cm^2^ and cultured in suspension for 16 h at 37 °C. Anoikis-resistant cells (AR) were isolated by depletion of dead cells using the Miltenyi Dead Cell Removal kit. For GSK591 studies, monolayer cells were treated at a final concentration of 500 nM for three consecutive days prior to AR assay. Cisplatin treatments were conducted in universal tubes for 2 h at 37 °C prior to seeding onto poly-HEMA-coated dishes. The presence of GSK591 was constant throughout all experiments including during pulse-treatments with cisplatin. For ionising radiation (IR) exposure, BCSCs were seeded onto poly-HEMA-coated dishes.

### Mammosphere formation assay

Conducted as described in [15].

### Isolation of CD24^low^/CD44^+^ BCSCs

Cells were incubated for 1 hr at 4 °C in the dark with FACS buffer (PBS-5% (v/v) FBS and 2 mM EDTA) containing either PE-conjugated anti-CD24, APC-conjugated anti-CD44 or relevant isotype controls (Miltenyi). Cells were sorted under pre-chilled sterile conditions into ice-cold PBS using a BD FACSAria Fusion flow cytometer (BD FACSDiva acquisition software v9.0).

### RNA-sequencing, splicing and gene expression analysis

MCF7 and AU565 cells were treated with GSK591 (500 nM) for three days followed by an AR assay in the continued presence of GSK591. Cells were then isolated for RNA-sequencing 16 h later (total 4 days GSK591 treatment). See Supplementary data for extended methods.

### Statistical analysis

The means of two independent groups were compared by a Student’s *t* test (two-sided, equal variance). Results are expressed as mean ± SEM or SD, and data were analysed by GraphPad Prism 9.2.0 and the Excel program of Microsoft Office.

## Supplementary information

Supplementary files

Supplementary Table 1

Supplementary Table 2

Supplementary Table 3

Supplementary Table 4

Supplementary Table 5

## Data Availability

Supplementary Figures, Tables and Methods are available in the Supplementary Figures and Tables file. RNA-seq from MCF7 bulk and AR BCSCs, and AU565 AR BCSCs has been deposited at the Gene Expression Omnibus, GEO study ID GSE261017.
