# Supplementary material for: PRMT5-regulated splicing of DNA repair genes drives chemoresistance in breast cancer stem cells
Source: Oncogene. 2024 Dec 18;44(13):862–76. doi: 10.1038/s41388-024-03264-1 (PMC11932929; doi:10.1038/s41388-024-03264-1)
Supplement: Supplementary file 1 — Supplementary files [file 41388_2024_3264_MOESM1_ESM.pdf]

PRMT5-regulated splicing of DNA repair genes drives chemoresistance in breast cancer stem cells

Matthew S. Gillespie[1,2,4], Kelly Chiang[1,4], Gemma L. Regan-Mochrie[1], Soo-Youn Choi[1], Ciara M. Ward[1], Debashish Sahay[1, 3], Paloma Garcia[1], Roland Arnold[1] and Clare C Davies[1*]

[1] Institute of Cancer and Genomic Sciences, University of Birmingham, B15 2TT, UK

[2] Present address: School of Cancer Sciences, University of Southampton, SO16 6YD, UK

[3] Present address: Johnson & Johnson, 1400 McKean Rd, Spring House, Pennsylvania 19002, USA

[4] These authors contributed equally

* Corresponding author: c.c.davies@bham.ac.uk

SUPPLEMENTARY DATA, FIGURE LEGENDS and METHODS

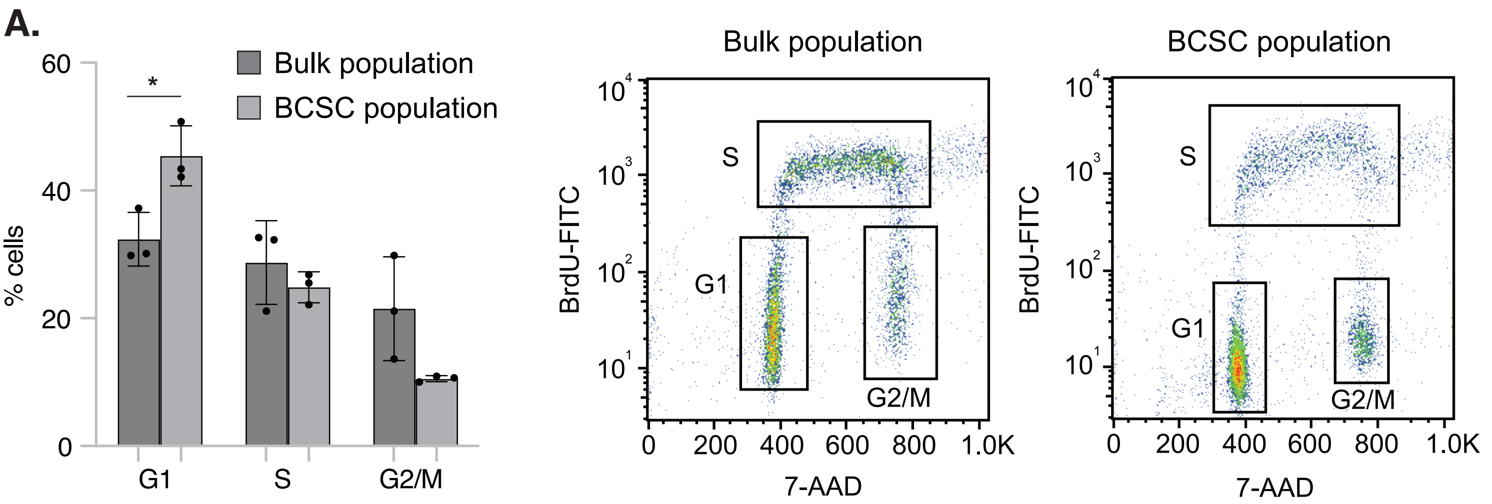

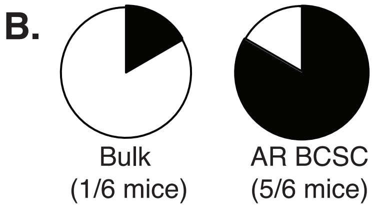

Gillespie et al: Supplementary Figure 1

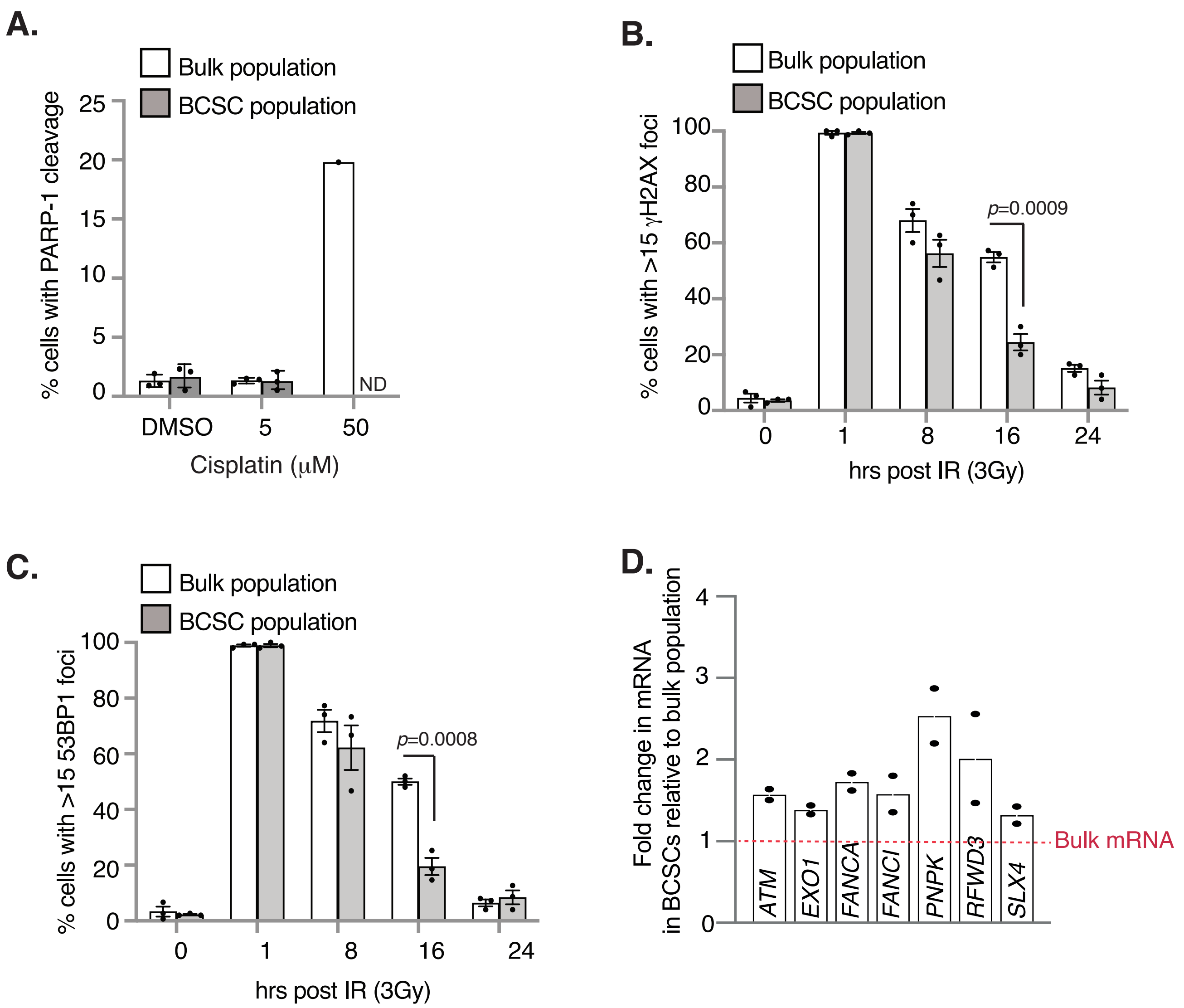

Gillespie et al: Supplementary Figure 2

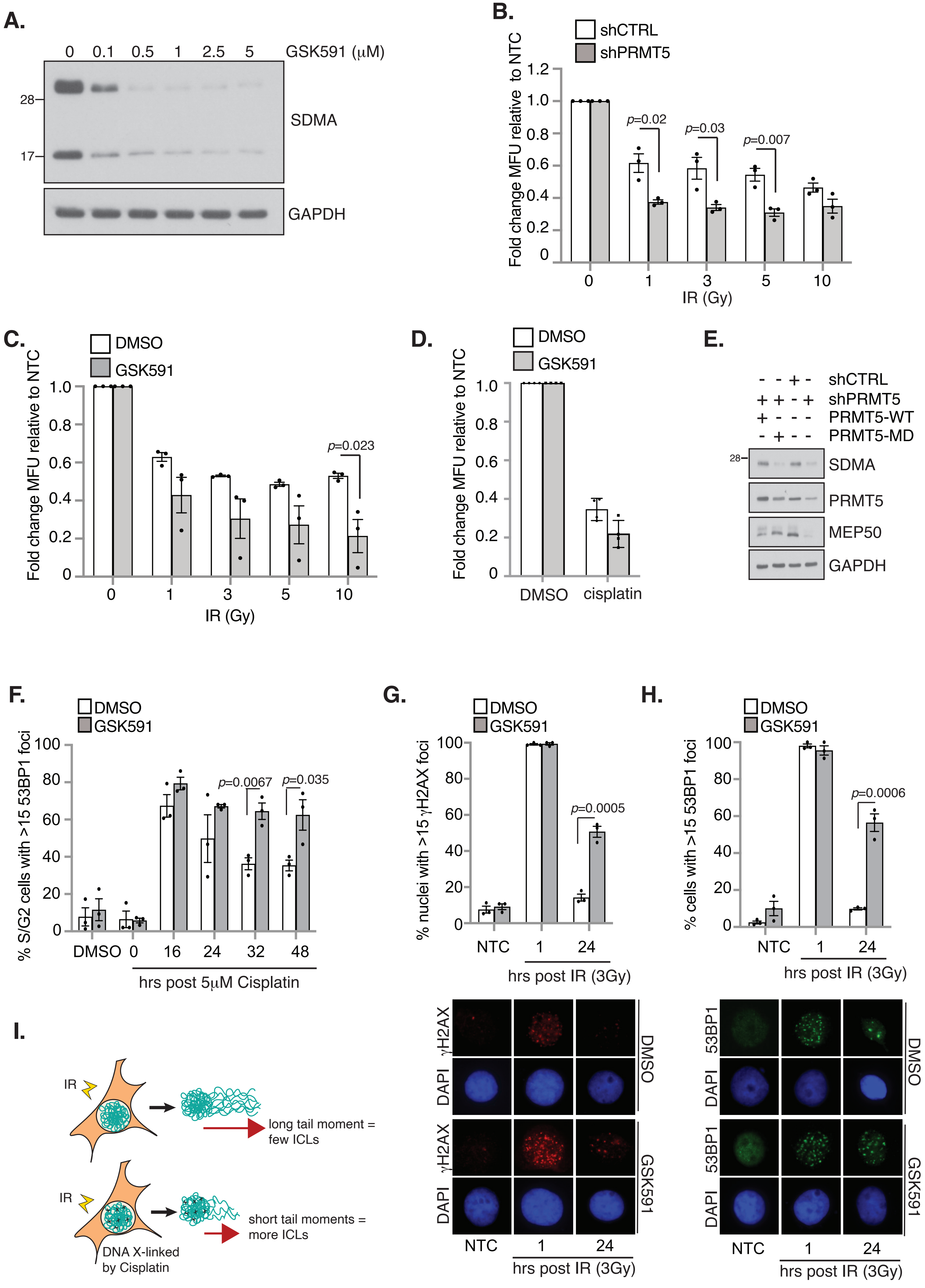

Gillespie et al: Supplementary Figure 3

A.

A3SS	A5SS	SE	RI
NABP1	*ATM	ALKBH3	ATR
PNKP	RAD9A	CENPX	DDX11
RPAIN		CHD1L	EME2
		EP400	FAN1
		EXO1	HDAC10
		FANCI	INO80E
		HDAC10	INTS3
		HSF1	MUTYH
		NPM1	PNKP
		PARPBP	POLD1
		RAD51AP1	RPAIN
		RAD51D	SIRT7
		RPAIN	WRAP53
		TREX2	
		USP10	
		USP28	

*validated as retained intron

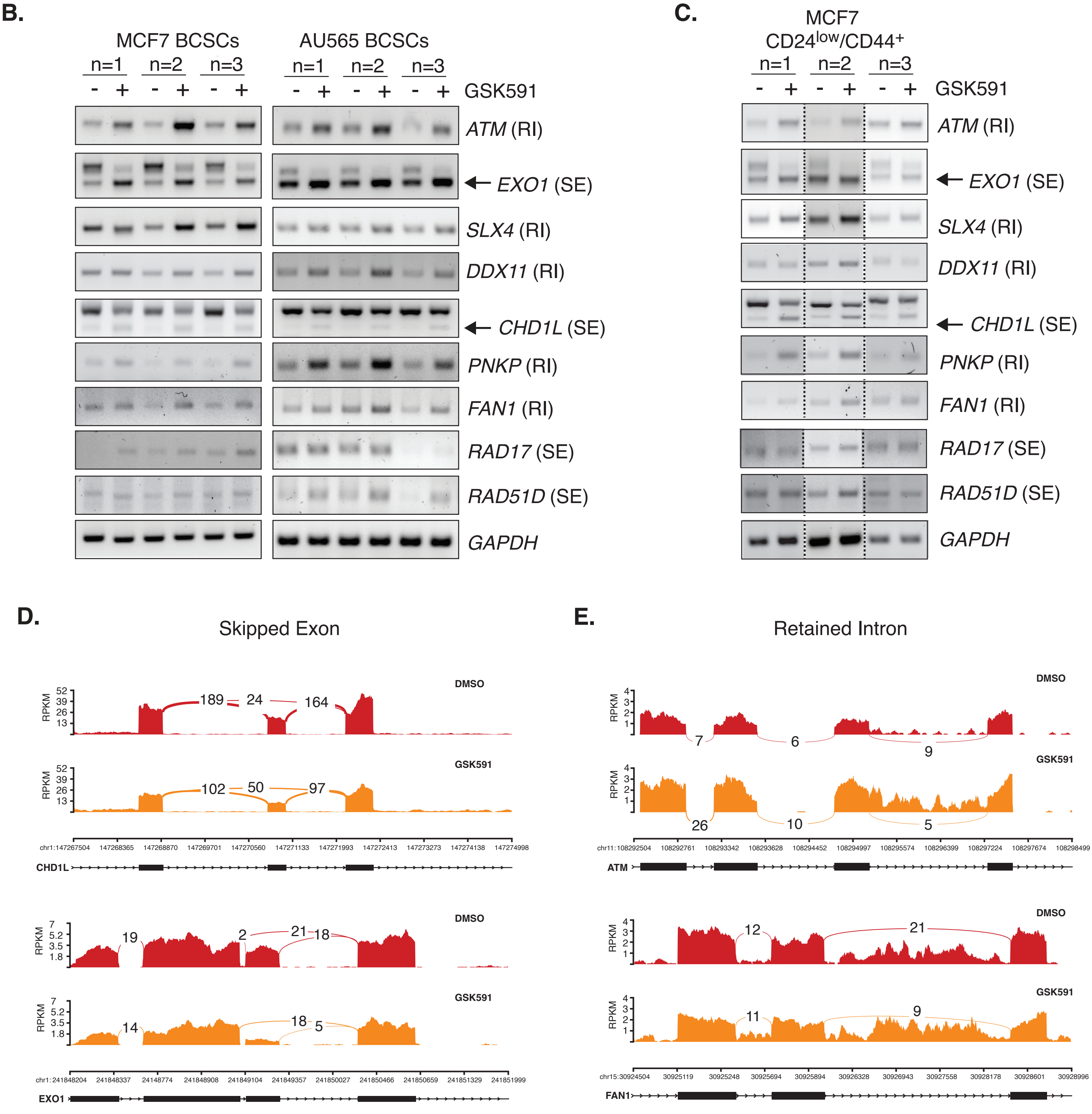

Gillespie et al: Supplementary Figure 4

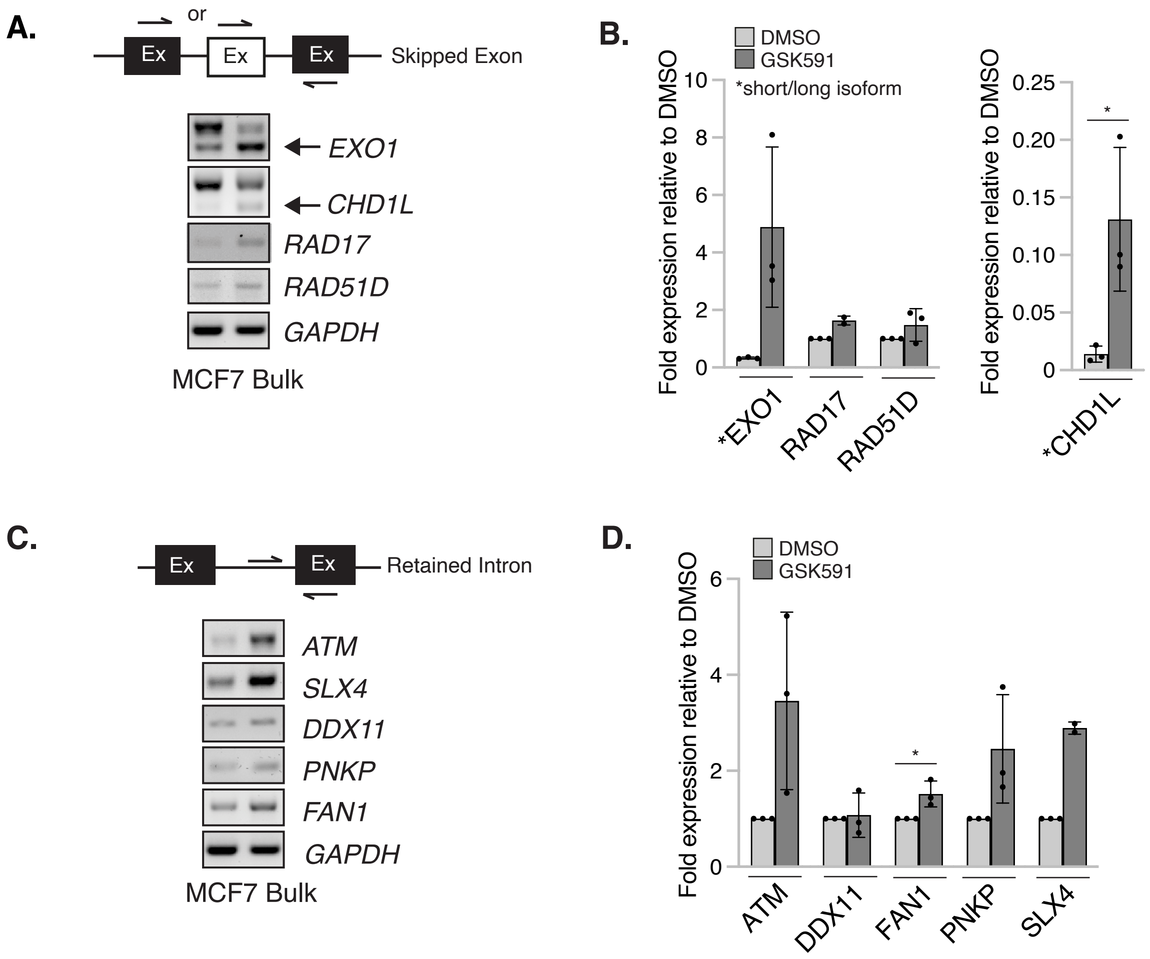
A.
or
Ex
Ex
Ex
Skipped Exon
EXO1
CHD1L
RAD17
RAD51D
GAPDH
MCF7 Bulk
B.
DMSO
GSK591
*short/long isoform
Fold expression relative to DMSO
*EXO1
RAD17
RAD51D
*CHD1L
C.
Ex
Ex
Retained Intron
ATM
SLX4
DDX11
PNKP
FAN1
GAPDH
MCF7 Bulk
D.
DMSO
GSK591
Fold expression relative to DMSO
ATM
DDX11
FAN1
PNKP
SLX4

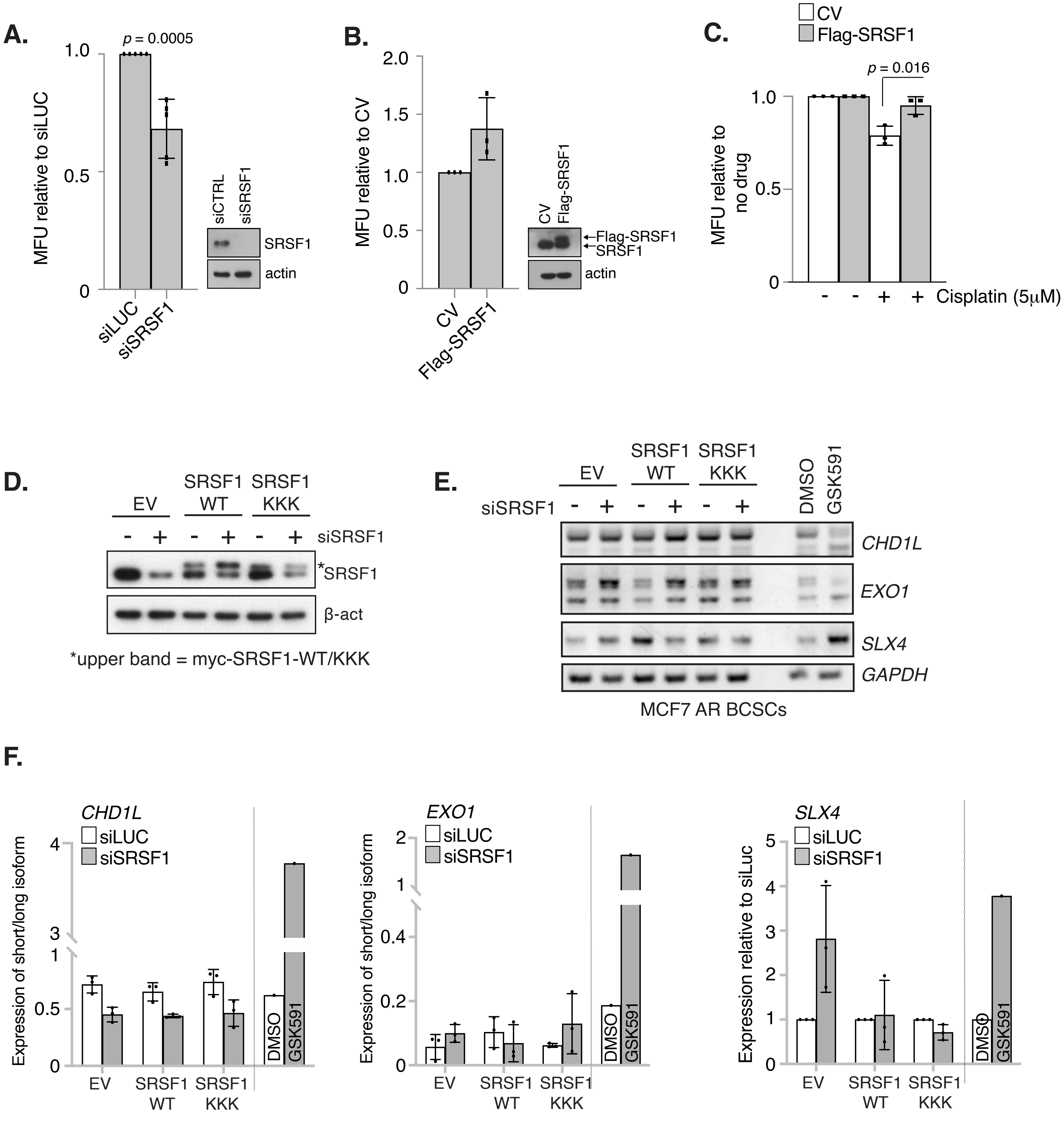

Gillespie et al: Supplementary Figure 6

SUPPLEMENTARY FIGURE LEGENDS

Supplementary Figure 1: Characterisation of AR isolated-MCF7 BCSCs

(A) BrdU pulse cell cycle analysis of MCF7 bulk and AR BCSCs (n=3; mean ± SD). Representative flow cytometry profiles are depicted. (B) Bulk or MCF7 AR BCSCs were implanted into NSG mice and tumour occurrence determined 33 days post-engraftment.

Supplementary Figure 2: MCF7 AR BCSCs are more radioresistant, exhibit increased DNA repair efficiency, and express higher levels of DDR-related genes

(A) PARP1 cleavage assay of AR BCSCs after cisplatin (mean ± SD). (B) γH2AX and (C) 53BP1 foci analysis of IR-treated AR BCSCs isolated from GSK591-treated MCF7 cells. (D) qPCR analysis of DDR gene expression in MCF7 bulk cells and AR BCSCs (n=2). Unless otherwise stated n=3; mean ± SEM; Student's *t*-test (two-sided, equal variance).

Supplementary Figure 3: PRMT5 regulates DNA repair in BCSCs following cisplatin and IR-induced DNA damage

(A) Immunoblot of SDMA levels in GSK591-treated MCF7 cells. Mammosphere assay of IR-treated (B) MCF7 shCTRL or shPRMT5 cells or (C) GSK591-treated MCF7 cells. (D) Mammosphere assay of cisplatin-treated (5μM) AU565 cells, with and without GSK591 (500nM). n=4; mean ± SD; Student's *t*-test (two-sided, equal variance). (E) Immunoblot of cell lines in Figure 2C. MD = methyltransferase dead. (F) 53BP1 foci analysis of GSK591 and cisplatin-treated MCF7 $CD24^{low}/CD44^{+}$ BCSCs. (G) γH2AX and (H) 53BP1 foci analysis in GSK591-treated MCF7 AR BCSCs after IR. (I) Schematic of alkaline comet assay. ICLs physically crosslink the DNA duplex; the extent of DNA ICL damage is determined by the inability of high-dose IR to fragment DNA. A high percentage of DNA within the tail signifies fewer ICLs whilst a low percentage tail moment indicates more ICLs. Unless otherwise stated n=3; mean ± SEM; Student's *t*-test (two-sided, equal variance).

Supplementary Figure 4: GSK591 promotes differential splicing of DNA damage response genes in BCSCs

(A) Table of common DSGs between MCF7 and AU565 AR BCSCs. Type of splicing event is indicated. Data taken from Supplementary Table 1. (B) Raw RT-PCR images of the three independent biological replicates for validation of GSK591-mediated splicing events MCF7 and AU565 AR BCSCs quantified in Figures 5B and 5D. In some cases, DMSO-treated signal was at background levels resulting in an artificially high enrichment in the GSK591-treated sample and thus omitted from quantitative analysis presented in Figure 5. (C) Raw RT-PCR images of the three independent biological replicates of GSK591-mediated splicing events in

CD24^{low}/CD44^{+}-isolated BCSCs quantified in Figure 5E-F. (D) Sashimi plots of RT-PCR-validated skipped exon events for *CHD1L* and *EXO1*. (E) Sashimi plots of the RT-PCR-validated intron retention events, for *ATM* and *FAN1*.

Supplementary Figure 5: Validation of PRMT5-dependent splicing events in bulk MCF7 cells (A) RT-PCR validation of SE splicing events in GSK591-treated bulk MCF7 cells. Representative image of n=3. (B) Densitometry quantification of data in (A). (C) RT-PCR validation of RI splicing events in GSK591-treated bulk MCF7 cells. Representative image of n=3. (D) Densitometry quantification of data in (C). Unless otherwise stated n=3; mean ± SD; Student's *t*-test (two-sided, equal variance).

Supplementary Figure 6: Methyl-deficient SRSF1 does not phenocopy GSK591 in the deregulation of *EXO1, CHD1L,* and *SLX4* splicing in MCF7 BCSCs.
(A) Mammosphere assay of MCF7 cells after siSRSF1. (B) Mammosphere assay of MCF7 cells overexpressing Flag-SRSF1. (C) Mammosphere assay of cisplatin-treated MCF7 cells overexpressing Flag-SRSF1, CV = control vector. (D-F) Alternative splicing analysis after SRSF1 depletion and reconstitution with WT or methyl-deficient (KKK) SRSF1 in MCF7 AR BCSCs. (D) Immuoblot analysis after siSRSF1 in bulk MCF7 cells stably expressing SRSF1-WT or SRSF1-KKK. (E) RT-PCR analysis of SE and RI events in MCF7 AR cells. n=3 for SRSF1 experiment; n=1 for GSK591 treatment. (F) Quantification of RT-PCR results shown in (E). mean ± SD.

SUPPLEMENTARY TABLES

Supplementary Table 1: List of differentially spliced genes identified within the GO term "DNA repair" in MCF7 and AU565 AR-isolated BCSCs.

Supplementary Table 2: Raw quantification values of RT-PCR images in Supplementary Figure 4 contributing to data presented in Figure 5.

Supplementary Table 3: Differentially expressed genes in MCF7 AR-isolated BCSCs after 4 days GSK591 (500nM).

Supplementary Table 4: qPCR data of gene expression after 4 days GSK591 (500nM) in MCF7 AR-isolated BCSCs, AU565 AR-isolated BCSCs and MCF7 CD24^{low}/CD44^{+}-isolated BCSCs.

Supplementary Table 5: Primers used in this study.

SUPPLEMENTARY MATERIALS AND METHODS

Colony forming assay

Bulk cells and AR BCSCs were seeded at a low cell density (in triplicate) onto 60mm dishes 24 hrs prior to treatment with IR. For cisplatin, both cell populations were treated in a universal tube for 2 hr at 37°C, washed twice with PBS and seeded onto 60mm dishes. Growth medium was replaced every 2-3 days and colonies fixed and stained with 1% (w/v) methylene blue (Merck Life Sciences) in 50% (v/v) ethanol after 14 days in culture.

5-bromo-2-deoxyuridine (BrdU) incorporation assay

Monolayer cells and AR BCSCs were incubated with 50μM BrdU (Merck Life Sciences) for 2 hrs at 37°C, fixed and permeabilised with 70% (v/v) ice-cold ethanol. Cell cycle was analysed using the FITC Mouse Anti-BrdU kit (BD Biosciences). All samples were counterstained with 12μg/ml 7-Aminoactinomycin D (7-AAD; Invitrogen). Flow cytometry was performed on a BD LSRFortessa X-20 equipped with BD FACSDiva acquisition software v9.0. Results were analysed using FlowJo software v10.6.1.

PARP-1 cleavage apoptosis assay

This was conducted using the apoptosis FACS assay kit (BD Biosciences). For $CD24^{low}/CD44^{+}$ BCSCs, antigen staining was carried out as described earlier using CD24-PE-Vio770 and CD44-APC (Miltenyi), prior to fixing. Stained cells were analysed with a BD LSRFortessa X-20 (BD FACSDiva acquisition software v9.0). Results were analysed using FlowJo software v10.6.1.

Immunocytochemistry

Cells were fixed in ice-cold 4% (w/v) paraformaldehyde (Merck Life Sciences) for 13 min on ice. Fixed cells were washed three times in ice-cold PBS and resuspended in PBS. A maximum of 60,000 cells were centrifuged (136 × g, 5min) onto a 6mm-diameter circular area on microscopic slides using a Shandon CytoSpin cytocentrifuge (Thermo Scientific). Slides were allowed to air dry for 15 min before treatment with ice-cold cytoskeletal pre-extraction buffer (PBS-0.5% Triton X-100 containing 10mM PIPES (pH 6.0), 300mM Sucrose, 100mM NaCl and 3mM $MgCl_2$) for 4 min at room temperature. After two rinses with ice-cold PBS, cells were permeabilised with PBS-0.5% (v/v) Triton X-100 for 5 min at room temperature, washed with ice-cold PBS (3 × 5 min), and blocked with PBS/5% FBS/1% bovine serum albumin/0.3% Triton X-100 for 1 hr at room temperature. Cells were incubated in a humid chamber at 4°C overnight with the primary antibody diluted in blocking buffer. Slides were washed with PBS-0.1% Tween (3 × 2 min), incubated with the relevant fluorophore-conjugated secondary

antibody diluted with blocking buffer for 1 hr at room temperature in the dark, washed with PBS-0.1% Tween (3 × 2 min) and nuclei counterstained using ProLong Gold Antifade Mountant containing 4′,6-diamidino-2-phenylindole (DAPI) (Invitrogen). Nuclear foci were counted by eye (150 nuclei/sample) using a Leica DM6000B upright widefield epifluorescence microscope interfaced with LAS X v3.4.18368.2 (Leica Microsystems).

Alkaline comet assay

The Comet Assay reagent kit (Bio-Techne) was used according to the manufacturer's instructions. Briefly, 50,000 cells were treated with or without cisplatin for 24 hrs, exposed to 30Gy IR. and then mixed with low melting agarose before being immediately layered onto CometSlides. The slides were immersed in pre-chilled lysis solution for 30 min at 4°C and transferred to an alkaline solution of pH>13 (300mM NaOH, 200mM EDTA) for 30 min at room temperature in the dark. Slides were then placed in an electrophoresis unit and subjected to an electric field of 1V/cm for 25 min in fresh alkaline electrophoresis buffer (300mM NaOH, 1mM EDTA) at 4°C. Following a wash with deionised water (3 × 3 min), the comet assay gels were dehydrated in 70% ethanol for 3 min and allowed to dry at room temperature in the dark. The DNA was stained by SYBR Safe DNA gel stain (1:1000) for 10 min and the remaining DNA stain washed off with deionised water. Stained cells were imaged using a Zeiss Axiovision 3 immunofluorescent microscope and images were obtained using ZEN Blue software (v2.3). A minimum of 50 cells/slide were counted and quantification of the percentage of DNA within the comet tail was carried out using the TriTek CometScore Freeware v1.5 software package (TriTek Corporation).

Immunoblotting

Cell lysates were prepared by sonicating in ice-cold 0.2% IGEPAL lysis buffer (150mM NaCl, 20mM Tris-HCl (pH 7.5), 0.5mM EDTA supplemented with 1mM Na_3VO_4, 50mM NaF, 1mM β-glycerophosphate, 100μM PMSF, 10μg/ml aprotinin and 10μg/ml leupeptin) and clarified by centrifugation. Proteins were separated by SDS-PAGE and transferred to PVDF membrane, blocked and incubated overnight in primary antibody. Membranes were washed and incubated with secondary antibody and visualised by enhanced chemiluminescence and X-ray film.

Tumour xenografts

Animal experiments were conducted in accordance with United Kingdom Home Office regulations. The tumour-initiating capacity of monolayer MCF7 cells and AR BCSCs was examined by xenotransplantation of cells into 4–6-week-old female NOD/Shi-scid/IL-2Rγ-null mice. Engraftment of human cells into mice was carried out as described [1]. For comparison of tumour initiation between bulk and BCSCs, anaesthetised mice were inoculated

subcutaneously into two contralateral flank sites with 50,000 monolayer cells or BCSCs diluted 1:1 in a mixture of DMEM: Matrigel (BD Biosciences) to a final injection volume of 200μl. To analyse the effects of GSK591 and cisplatin on BCSCs *ex vivo*, $1x10^6$ MCF7 cells were implanted as a 1:1 cell:Matrigel mix. To support engraftment, a small subcutaneous incision close to the base of the tail was made and a 90-day slow-release oestradiol pellet (0.72mg) (Innovative Research of America) implanted enabling continuous delivery of hormones. All subsequent handling and operations were carried out in a laminar air flow cabinet to further reduce the risk of infection. Tumour size was determined using callipers every two days and mice were sacrificed at day 49-post injection. For mammosphere assays performed on MCF7 cells derived from xenografts, tumours were dissociated using the Miltenyi Tumour Cell Dissociation kit (Miltenyi) according to manufacturer's instructions. Cells were seeded at 5000 cells/well, treated with GSK591 and/or cisplatin, and scored for both primary and secondary mammospheres and results expressed as MFU.

RNA-sequencing, splicing and gene expression analysis

RNA libraries were prepared using the NEBNext® Ultra™ II DNA Library Prep Kit for Illumina and polyA-selected prior to sequencing on the NextSeq sequencer. Raw RNA reads were aligned to hg38 using STAR [2]. Using the Galaxy web platform usegalaxy.org (The Galaxy Community, 2022 update), read count files were generated using the featureCounts tool [3] and differential gene expression was performed by DESeq2 [4], with a threshold of p-adj<0.05 and fold change >50%. Gene ontology analysis was performed using DAVID (version 2021, v2023q4) [5,6]. Differential splicing analysis was performed using rMATS Turbo 4.1.1 [7–9], after aligning to the Reference Genome (Ensembl CHR38, Gene Annotation 104) using STAR [2] version 2.72 (parameters: --twopassMode Basic --alignIntronMin 20 --alignMatesGapMax 200000 --alignIntronMax 200000, --alignEndsType EndToEnd) with thresholds of FDR <0.1 and ΔPSI>0.1. Sashimi plots were generated using rmats2sashimiplot. Volcano plots were generated using ggplot2 (https://ggplot2.tidyverse.org.) using R Studio (http://www.rstudio.com/).

RNA extraction, qPCR and RT-PCR

RNA was extracted from cell pellets using the GenElute Mammalian Total RNA Isolation Kit (Merck Life Sciences) according to manufacturer's instructions. RNA was reverse transcribed using the SensiFAST™ cDNA Synthesis Kit (Bioline) according to manufacturer's instructions. qPCR was performed using SYBR® Green PCR Master Mix (Thermo Scientific) according to manufacturer's instructions. See Supplementary Table 5 for primers used in this study.

Key resources table

REAGENT or RESOURCE	SOURCE	IDENTIFIER
**Antibodies**		
53BP1	Novus Biologicals	NB100-304SS
Alexa Fluor 594 anti-Mouse	Invitrogen	A11032
β-actin-HRP	CST	5125
BrdU-FITC	BD Biosciences	556028
CD24-PE	Miltenyi	130-095-952
CD24-PE-Vio770	Miltenyi	130-108-35
CD44-APC	Miltenyi	130-095-17
CENPF	Abcam	ab5
Cleaved PARP-1-PE	BD Biosciences	51-9007684
DyLight 488 anti-Rabbit	Invitrogen	35552
FANCD2	Novus Biologicals	NB100-182
GAPDH	CST	2118
γH2AX	Millipore	05-636
Goat Anti-Rabbit IgG Antibody (H+L), Biotinylated	Vector Laboratories	BA-1000-1.5
MEP50	NEB	2823
Mitosin	BD Biosciences	610768
Mouse IgG1	CST	5415
Mouse IgG1-APC	Miltenyi	130-098-214
Mouse IgG1-FITC	BD Biosciences	556028
Mouse IgG1-PE	Miltenyi	130-092-212
Mouse IgG-HRP	Dako	P0399
PRMT5	Millipore	07-405
Rabbit IgG1	Millipore	12-370
Rabbit IgG-HRP	Dako	P0399
Rad51	Millipore	PC130
RPA	Millipore	NA18
SDMA	CST	13222
**Reagents and Chemicals**		
DMEM/F12 (without phenol red)	Gibco	21041025
B-27 Supplement minus vitamin A	ThermoFisher	12587010
Human recombinant epidermal growth factor	Miltenyi	130-097-750
Poly (2-hydroxyethyl methacrylate) (poly-HEMA)	Merck Life Sciences	P3932-25G
Cisplatin	Cambridge Biosciences	13119
GSK591	Merck Life Sciences	SML1751
ProLong Gold Antifade Mountant containing 4′,6-diamidino-2-phenylindole (DAPI)	Invitrogen	11569306
Methylene blue	Fisher Life Sciences	414240250
5-bromo-2-deoxyuridine (BrdU)	Merck Life Sciences	B5002-500MG
BD Growth Factor reduced Matrigel	BD Biosciences	354230

Oestradiol pellet	Innovative Research of America	NE-121
**Commercial assays/kits**		
Apoptosis, DNA Damage and Cell Proliferation Kit	BD Biosciences	2869407
FITC Mouse Anti-BrdU Set Kit	BD Biosciences	556028
GenElute Mammalian Total RNA Miniprep Kit	Merck Life Sciences	RTN70
Miltenyi Dead cell Removal Kit	Miltenyi	130-090-101
Comet Assay	Bio-Techne	4250-050-K
SensiFAST cDNA Synthesis Kit	Bioline	BIO65053
**Experimental models: Organisms/strains**		
Mouse: NOD/Shi-scid/IL-2Rg-null	Biomedical Services Unit (BMSU), University of Birmingham	N/A
**Oligonucleotides**		
RT-PCR and qPCR primer sequences	See Supplementary Table 5	N/A
**Software and algorithms**		
Leica Application Suite X (LAS X; v3.4.18368.2)	Leica Microsystems	https://www.leica-microsystems.com
BD FACSDiva (v9.0)	BD Biosciences	https://www.bdbiosciences.com
FlowJo (v10.6.1)	BD Biosciences	https://www.flowjo.com
MxPro qPCR Software	Agilent Technologies	https://www.agilent.com
FASTQC (v0.11.9)	Andrews, 2010	https://www.bioinformatics.babraham.ac.uk
STAR (v2.7.0a)	Dobin *et al.*, 2012	https://github.com/alexdobin/STAR
Salmon (v1.8.0)	Patro *et al.*, 2017	https://combine-lab.github.io/salmon
Galaxy (v22.01.rc1)	Johns Hopkins University	https://usegalaxy.org
DESeq2 (v1.34.0)	Love *et al.*, 2014	https://github.com/thelovelab/DESeq2
DAVID (version 2021, v2023q4)	Huang *et al.*, 2009	https://david.ncifcrf.gov
Multivariate Analysis of Transcript Splicing with Replicates (rMATS; 4.0.2)	Shen *et al.*, 2014	https://rnaseq-mats.sourceforge.io
GraphPad Prism (v9.2.0)	Dotmatics	https://www.graphpad.com/features
Microsoft Excel (v2013)	Microsoft	https://www.microsoft.com/en-gb/microsoft-365/excel
ggplot2	Wickham H (2016). ISBN 978-3-319-24277-4	https://ggplot2.tidyverse.org
RStudio	Posit, PBC	https://www.R-project.org/

Cell lines		
MCF7 cells	ATCC	HTB-22
T47D cells	ATCC	HTB-133
AU565 cells	ATCC	CRL-2351
MCF7-shCTRL	In-house	[1]
MCF7-shPRMT5	In-house	[1]
MCF7-shPRMT5/pHIVzsGreen-PRMT5/pHIV-dTOM-MEP50	In-house	[1]
MCF7-shPRMT5/pHIVzsGreen-PRMT5 (G367A/R368A)/pHIV-dTOM-MEP50	In-house	[1]

REFERENCES

1 Chiang K, Zielinska AE, Shaaban AM, Sanchez-Bailon MP, Jarrold J, Clarke TL *et al.* PRMT5 Is a Critical Regulator of Breast Cancer Stem Cell Function via Histone Methylation and FOXP1 Expression. *Cell reports* 2017; **21**: 3498–3513.

2 Dobin A, Davis CA, Schlesinger F, Drenkow J, Zaleski C, Jha S *et al.* STAR: ultrafast universal RNA-seq aligner. *Bioinformatics* 2013; **29**: 15–21.

3 Liao Y, Smyth GK, Shi W. featureCounts: an efficient general purpose program for assigning sequence reads to genomic features. *Bioinformatics* 2014; **30**: 923–930.

4 Love MI, Huber W, Anders S. Moderated estimation of fold change and dispersion for RNA-seq data with DESeq2. *Genome Biol* 2014; **15**: 550.

5 Sherman BT, Hao M, Qiu J, Jiao X, Baseler MW, Lane HC *et al.* DAVID: a web server for functional enrichment analysis and functional annotation of gene lists (2021 update). *Nucleic Acids Res* 2022; **50**: W216–W221.

6 Huang DW, Sherman BT, Lempicki RA. Systematic and integrative analysis of large gene lists using DAVID bioinformatics resources. *Nat Protoc* 2009; **4**: 44–57.

7 Shen S, Park JW, Lu Z, Lin L, Henry MD, Wu YN *et al.* rMATS: Robust and flexible detection of differential alternative splicing from replicate RNA-Seq data. *Proc National Acad Sci* 2014; **111**: E5593–E5601.

8 Park JW, Tokheim C, Shen S, Xing Y. Deep Sequencing Data Analysis. *Methods Mol Biol* 2013; **1038**: 171–179.

9 Shen S, Park JW, Huang J, Dittmar KA, Lu Z, Zhou Q *et al.* MATS: a Bayesian framework for flexible detection of differential alternative splicing from RNA-Seq data. *Nucleic Acids Res* 2012; **40**: e61–e61.
